# Secondhand fashion consumers exhibit fast fashion behaviors despite sustainability narratives

**DOI:** 10.1038/s41598-025-19089-1

**Published:** 2025-10-07

**Authors:** Meital Peleg Mizrachi, Ori Sharon

**Affiliations:** 1 Department of Economics - the Economic Growth Center, Yale Center for Business and Environment, Yale University, New Haven, CT 06511 USA; 2 Faculty of Law and Environmental Regulation Program, Bar Ilan University, Ramat Gan, 5290002 Israel

**Keywords:** Sustainable fashion, Second-Hand clothing, Rebound effect, Moral licensing, Overconsumption., Sustainability, Psychology and behaviour

## Abstract

The fashion industry contributes 2–8% of global greenhouse gas emissions, driven by rising clothing consumption and the proliferation of fast fashion. Fast fashion accelerates environmental harm through rapid production cycles, low costs, and short garment lifespans. Secondhand clothing markets are often promoted as a sustainable alternative, promising extended use and reduced waste. These markets have grown rapidly, with global sales reaching $177 billion in 2022 and projected to double by 2027. Despite this growth, few studies have empirically examined whether secondhand purchasing displaces or merely supplements primary market consumption. This study addresses that gap using a nationally representative survey of 1,009 U.S. consumers. We find that secondhand consumption is positively correlated with new clothing purchases (*r* = 0.58, *p* < 0.01), particularly among younger consumers and frequent shoppers. Cluster and principal component analyses reveal that highly engaged secondhand consumers also exhibit high overall consumption and short garment retention. Although many report high sustainability knowledge, such knowledge does not reliably predict sustainable behavior. Drawing on rebound and moral licensing theories, we suggest that secondhand purchases may psychologically or economically justify continued overconsumption. These findings challenge assumptions about resale’s environmental benefits and support policy interventions to realign resale practices with sustainability goals.

**Supplementary Information:**

The online version contains supplementary material available at https://doi.org/10.1038/s41598-025-19089-1.

## Introduction

### Rebound or reduction?? The sustainability promise and pitfalls of secondhand fashion

The fashion industry ranks among the most environmentally damaging sectors globally^1–4^. Amid growing concerns over its environmental and social impacts, secondhand markets have emerged as a purportedly sustainable alternative. Promoted by fashion brands, sustainability-oriented firms, consumers, and policymakers alike, resale platforms and garment collection programs are increasingly framed as tools for reducing waste and extending product lifespans within circular economy frameworks^5^.

Despite this momentum, the environmental efficacy of secondhand fashion remains underexamined. Although resale markets have expanded rapidly^6^it is unclear whether secondhand purchases displace new clothing consumption or merely supplement it^7^. If the latter, resale might inadvertently reinforce overconsumption rather than mitigate it. Behavioral theories such as the rebound effect and moral licensing complicate the sustainability promise of secondhand fashion. These frameworks suggest that perceived ethical consumption (e.g., buying used clothing) may paradoxically lead to increased overall consumption by justifying additional purchases or alleviating guilt^8–17^.

While such behavioral insights are gaining traction in sustainability research, their application to the fashion sector generally, and to secondhand consumption specifically, remains limited. This study addresses that gap by examining how secondhand clothing trends interact with broader fashion consumption patterns. In contrast to studies that emphasize stated intentions or responses to marketing cues^8,17^our analysis focuses on actual purchasing and disposal behaviors across both primary and secondary markets using a large, nationally representative sample of U.S. consumers. Our analysis investigates whether sustainability-oriented attitudes toward secondhand fashion are associated with general reductions in clothing purchases, or whether they give rise to behavioral spillovers that undercut environmental gains. We explore these dynamics across market segments, product categories, and demographic groups to clarify how behaviors perceived as sustainable may, in practice, be embedded within broader systems of high-volume consumption. In doing so, we contribute to a more empirically grounded understanding of whether secondhand fashion disrupts—or merely reproduces—the logics of fast fashion.

### Literature review and theoretical framework

#### Fast fashion: structural logics and environmental costs

The fashion industry ranks among the largest global polluters, contributing an estimated 2–8% of greenhouse gas emissions^18^–more than international flights and maritime shipping combined^19^. Estimates vary depending on methodological scope, with some including only production while others factor in emissions from transport and consumer use. This variation underscores the complexity of assessing the industry’s climate impact but affirms its status as a major contributor to global emissions.

Systemic overproduction, and a consumer culture that prioritizes trends, affordability, and convenience over sustainability, accelerate environmental and social tolls^20–22^. Textile production uses 93 billion cubic meters of water annually, and dyeing and finishing processes are the second-largest source of freshwater pollution globally^1–4^. These harms are concentrated in low- and middle-income countries, where production is outsourced to minimize costs. In manufacturing hubs such as Bangladesh, India, and Cambodia, untreated wastewater from dyeing facilities contaminates rivers with heavy metals, azo dyes, and microplastics, compromising drinking water, agriculture, and aquatic biodiversity^23^. These environmental harms are accompanied by social injustices, including wage theft, union suppression, unsafe working conditions, and gender-based violence against women^24–27^.

While these systemic issues pervade the global fashion supply chain, they are intensified by the rise of fast fashion, a model defined by rapid design, production, and distribution cycles that enable brands to release new collections within weeks or even days^28,29^. Over the past two decades, this model has nearly doubled global garment production and driven an estimated 400% increase in clothing consumption^30,31^. Fast fashion faces growing criticism for both upstream harms (e.g., labor abuses and resource depletion)^8^ and downstream effects, including massive volumes of textile waste. In 2023 alone, the industry produced an estimated 2.5 to 5 billion surplus garments, valued at $70–140 billion, many of which ended up in landfills or incinerators^8^.

#### Limitations and contradictions in secondhand fashion markets

A significant share of this surplus enters global secondhand markets through donation streams. Although donating used clothing is widely perceived in high-income countries as a sustainable practice, the volume of overproduction has overwhelmed charitable systems^32^. Large quantities are exported to the Global South, where they saturate local markets, suppress domestic production, and contribute to what scholars term “waste colonization”^21,33^. Efforts to limit these imports, such as the East African Community’s proposed 2016 ban, have faced political and economic pushback^34^. This convergence of overproduction and donation-driven saturation illustrates the limits of secondhand redistribution as a sustainability strategy. Rather than mitigating upstream harms, the global overflow of used clothing may exacerbate inequalities and perpetuate unsustainable production volumes.

In response to growing scrutiny, fast-fashion brands have launched secondhand initiatives as part of their sustainability strategies. Fast and ultra-fast fashion brands such as H&M, Shein, and Zara now promote resale platforms and garment collection programs as efforts to reduce waste. These efforts are situated within the broader discourse of the circular economy^35^—a production and consumption model that aims to minimize waste, retain the value of products and materials for as long as possible, and regenerate natural systems through reuse, repair, refurbishment, and recycling^36^.

The secondhand market is frequently championed for its potential to reduce textile waste, extend product lifespans, and promote circularity in the fashion sector^37–39^. Major policy initiatives, including the European Union’s Strategy for Sustainable and Circular Textiles and recommendations from the United Nations Environment Program, promote resale as a solution to overproduction and overconsumption^38,40–42^. From 2021 to 2022, the global secondhand market grew by 28%, with resale projected to reach $350 billion by 2027^6^. This growth reflects shifting consumer preferences, digital innovation, brand positioning, and regulatory support, all advancing the narrative that resale can anchor sustainable fashion.

Yet, practices in secondary markets often fall short of these ambitions. Rapid disposal of wearable clothing—driven by trend fatigue or space constraints—and aggressive marketing by major resale platforms may reinforce fast-fashion dynamics rather than disrupt them^43–45^. Critics question the environmental and social credibility of resale fashion, particularly when integrated into business models that maintain high production volumes. Some companies promote garment reuse while simultaneously accelerating new production cycles and contributing to textile waste^7^. These contradictions reflect a “sustainability bias,” where environmental virtue is projected rhetorically but not operationalized structurally^46^. Such practices suggest that secondhand initiatives primarily serve reputational aims rather than meaningful sustainability goals.

Empirical studies confirm that resale has not slowed activity in primary markets; Instead, both have expanded simultaneously. The secondhand market is forecasted to grow from $39 billion to $74 billion by 2029, yet primary market growth remains steady^6,47–49^. This parallel expansion casts doubt on the environmental benefit of resale and suggests that secondhand purchases may often supplement, rather than replace, conventional fashion consumption.

Taken together, these dynamics inform our first two hypotheses. We hypothesize (H1) that greater total clothing purchases, whether new or used, correlate with higher textile waste generation. We further hypothesize (H2) that secondhand consumption is positively correlated with primary market consumption, rather than displacing it. Both expectations reflect the concern that secondhand markets, while framed as sustainable, may reproduce rather than challenge fast fashion logics.

These hypotheses address a core empirical gap in sustainability research: whether secondhand purchasing offsets or merely adds to new clothing consumption. Clarifying this relationship is critical, as resale is increasingly promoted as a sustainable solution despite limited evidence that it curbs fast fashion demand.

#### Demographic factors driving fashion consumption

Understanding how different demographic groups engage with fashion, both new and secondhand, is essential for assessing the sustainability implications of consumption patterns. Age, gender, political orientation, and education all shape consumer values, motivations, and behaviors. This section examines how these factors influence participation in fast fashion and resale markets, with particular attention to whether engagement with secondhand fashion aligns with or contradicts broader sustainability goals.

##### Generational and Value-Based drivers of resale engagement

Generational trends play a crucial role in shaping engagement with secondhand fashion. Younger consumers—particularly Gen Z and Millennials—have leveraged digital platforms and social media to conduct secondhand transactions^50^. Luxury resale has also gained broader appeal^51^offering aspirational access to premium brands at reduced prices with authentication that signals quality and sustainability^50^. Studies indicate that both economic and environmental motivations influence participation in the secondhand market^52,53^while the rise of the sharing economy has further normalized resale behaviors^54,55^.

Importantly, the appeal of resale is shaped not only by sustainability concerns but also by broader consumer preferences. As Sumod, Mishra, and Rangnekar observe, “the normalization and glorification of buying secondhand fashion through celebrity endorsements and social influence have played a significant role in creating a consumer culture of secondhand clothing”^56^. Secondhand apparel is increasingly portrayed as fashionable, accessible, and morally superior. Yet this framing often coexists with continued overconsumption. As the same authors note, “many consumers want businesses to make sustainability a priority, [but] only some people are willing to pay the price for environmentally friendly products”^56^.

These observations highlight the contradictory messages that influence the attitudes and consumption patterns of younger generations. Digital platforms promoting constant novelty encourage both secondhand and fast fashion purchasing, making it essential to analyze resale within broader consumption systems rather than as an isolated sustainable practice.

Accordingly, we hypothesize (H3) that younger individuals will report higher frequency of new clothing purchases, and (H4) that they will report higher rates of secondhand clothing purchases compared to older individuals. These hypotheses address how generational differences shape dual engagement with resale and fast fashion, an important consideration for policy and marketing efforts targeting younger consumers, who may simultaneously encourage and undermine sustainability practices.

##### Gender and secondhand consumption

Consumer engagement with resale also reflects gendered preferences. Studies show that women are more likely to view secondhand shopping positively, associating it with creativity, fashionability, and enjoyment, while men often express concerns about hygiene, time investment, and social stigma^57^.

These attitudinal differences reflect broader trends in sustainable consumption, where women are generally more inclined to express environmental concern, support ethical brands, and adopt alternative consumption practices^58^. These tendencies may stem from gendered socialization processes that shape perceptions of environmental responsibility and fashion behaviors.

In line with this literature, we hypothesize (H5) that women will purchase more secondhand clothing than men, and (H6) that women will report more positive attitudes toward sustainable fashion than men. These hypotheses reflect the expectation that gendered norms and values influence engagement with resale and sustainable fashion more broadly.

Importantly, these hypotheses also aim to explore whether women’s higher engagement with secondhand fashion reflects deeper sustainability commitments or simply reproduces fast fashion consumption through alternative channels. Understanding this distinction is essential for assessing the gendered dimensions of sustainability and for informing more effective consumer-facing strategies.

##### Political and educational predictors of sustainable fashion behavior

While much of the sustainable fashion literature focuses on consumer preferences and values, emerging research highlights the role of political ideology and educational attainment in shaping consumption behavior^59^. Individuals with progressive political orientations are more likely to adopt environmentally responsible lifestyles, including participation in secondhand markets, whereas conservative consumers tend to prefer conventional consumption patterns^57,60^.

Similarly, higher levels of education and awareness about the fashion industry’s environmental and social harms are linked to greater engagement in sustainability-oriented behaviors^61^. Education often correlates with increased access to environmental information, stronger ethical commitments, and higher self-efficacy in adopting alternative behavioral practices^62^.

These ideological and cognitive predictors suggest that fashion consumption is not driven solely by price or style but is also embedded in broader worldviews. Political orientation and education serve as proxies for normative commitments, shaping how individuals interpret and respond to sustainability discourses.

Accordingly, we hypothesize (H7) that higher levels of education and awareness will be associated with lower consumption of new clothing, particularly at higher price points. We further hypothesize (H8) that politically engaged individuals will report higher levels of secondhand clothing purchases.

These hypotheses test whether sustainability behaviors reflect genuine normative commitments, or whether political orientation and education function primarily as markers of green identity. This distinction has important implications for the effectiveness of value-based policy appeals and targeted interventions.

#### Behavioral drivers: novelty, impulsivity, and return practices

Beyond demographic and ideological predictors, fashion consumption is shaped by behavioral trends linked to novelty-seeking and impulsivity. The abundance of inexpensive, unique items—combined with fear of missing out (“FOMO”)—encourages frequent purchasing in both primary and secondary markets^63,64^. Online platforms that update inventories daily, along with the popularity of thrift hauls, reinforce fast-fashion dynamics even within resale^65^.

This culture of immediacy contributes to impulsive buying, brief garment retention, and high return rates^66^particularly in fast fashion^67,68^. Return policies designed for convenience have normalized speculative shopping, where consumers purchase multiple items with the intention of returning some or most^69^.

These dynamics increasingly extend to resale markets, where low prices and fast-moving inventory encourage risk-free experimentation. Digital resale platforms mirror many conventional e-commerce practices, including personalized recommendations and frictionless returns, blurring behavioral distinction between secondhand and primary retail^70^. Returns have thus become an embedded component of contemporary fashion consumption, enabling consumers to churn through garments with minimal economic or psychological cost^70^.

Accordingly, we hypothesize (H9) that increased frequency and volume of new clothing purchases will be associated with higher return rates. This hypothesis addresses a gap in understanding how speculative shopping and return intersect with unsustainable purchasing patterns across both primary and secondary markets. Return rates may serve as behavioral indicators of overconsumption, even in contexts framed as lower-impact or circular.

#### Behavioral spillovers: rebound effects and moral licensing

While secondhand consumption is often framed as environmentally virtuous, behavioral research complicates this narrative. A key concern is that resale participation may trigger rebound effects or moral self-licensing, ultimately increasing overall consumption^8–10^. The rebound effect, rooted in environmental economics, describes a pattern in which perceived environmental or financial savings encourage further consumption, offsetting expected gains^10–13^. In the fashion context, consumers may use reduced costs or guilt from secondhand purchases to justify additional acquisitions.

Moral licensing theory complements this account. It suggests that engaging in a virtuous act, such as purchasing secondhand clothing, can create a sense of moral “credit” that allows consumers to justify subsequent unsustainable choices^14,15^. This mechanism is well-documented in consumer research and may explain why individuals who express strong sustainability values still engage in high-volume consumption^16^. Relatedly, Hall et al. (2018) argue that sustainable choices may serve expressive or identity-signaling purposes, rather than reflecting deeper behavioral commitments^17^.

These dynamics—though not well-examined in the literature on sustainable fashion—may grow more pronounced as resale becomes more mainstream. If consumers perceive secondhand purchases as morally compensatory, this could reduce guilt associated with continued or even increased fashion consumption, undermining resale’s environmental promise.

These insights inform our final hypothesis. We hypothesize (H10) that greater knowledge of the fashion industry’s environmental and social harms will be associated with more sustainable purchasing behavior. Nevertheless, we remain attentive to the possibility that knowledge may not consistently reduce consumption, particularly when mediated by moral licensing dynamics.

This hypothesis explores a key conflict in sustainability strategy: whether increased awareness leads to real behavioral change or merely acts as a psychological shield that allows unsustainable habits to continue. Clarifying this link is crucial, since consumer education remains a fundamental part of most sustainability efforts.

### Research questions and contribution

Building on the behavioral, demographic, and structural dynamics outlined above, this study empirically tests the relationship between secondhand fashion participation and broader consumption patterns. We examine whether engagement with resale markets acts as a substitute for, or rather a complement to, primary market consumption—and whether it advances or undermines sustainability goals.

Specifically, we explore the following research questions:

1. How do consumers engage with primary and secondary markets simultaneously in terms of purchase quantities, frequency, textile waste generation, and changes in purchasing behavior over time?
2. How do attitudes toward sustainability in fashion and secondhand clothing, as well as purchasing considerations, influence purchasing and disposal behaviors across markets?
3. How do demographic characteristics, including age, gender, income, employment status, and political engagement, relate to consumption patterns across markets?
4. To what extent does knowledge of the environmental and social harms of the fashion industry influence purchasing and disposal behaviors across markets?

These questions extend existing research on sustainability in fashion by integrating structural, ideological, and psychological mechanisms into a unified empirical framework. They also build on the contributions of Olson (2022) and Hall et al. (2018)^8,17^who demonstrated how sustainability messaging and moral identity signaling can influence, and often complicate, consumer behavior in the fashion sector. Olson finds that sustainability-oriented marketing in fast-fashion can trigger rebound effects, while Hall et al. explore the expressive and inconsistent nature of sustainability behaviors. Our study complements this work by focusing on observed consumer behavior rather than short-term self-reported intentions, emphasizing the secondary market as a site of sustainability signaling, and examining how primary and secondary market engagement interact across purchasing and disposal practices. It also empirically tests the relevance of rebound effects and moral licensing in the context of secondhand fashion.

## Methods

### General

This study investigates attitudes and behaviors related to the consumption of fashion products in the United States in the primary and secondary markets. Data were collected through an online survey conducted during two time periods: July 15–22, 2024, and August 31–September 15, 2024. The survey was administered by Centiment Company using pre-recruited online survey panel providers. Standard panel management and quality assurance procedures were followed to ensure data reliability. The sample was balanced by gender, and participants received small monetary rewards redeemable through an online payment service.

To maintain data integrity, a loyalty check was embedded within the questionnaire. Respondents who failed this check were excluded from the sample.

Recognizing the potential biases inherent in online panel surveys, such as self-selection and overrepresentation of certain groups, several measures were implemented to enhance the validity and generalizability of the findings. Random sampling techniques ensured that all individuals in the target population had an equal chance of selection. Diverse recruitment strategies-including outreach via email and social media platforms-were employed to capture a broader and more representative participant base. Monetary incentives were carefully structured to encourage participation across a wide demographic spectrum, minimizing the likelihood of response biases favoring highly motivated individuals.

### Questionnaire design

The questionnaire was developed by the research team, informed by prior studies in the field, to examine various aspects of fashion consumption and sustainability:

- Fashion Purchasing Habits: Frequency and quantity of purchases were assessed using items adapted from Weber and Weber (2021)^71^.
- Environmental Knowledge and Approaches to Sustainable Fashion: Items were based on frameworks from Colasante and D’Adamo (2021) and Martinez and Wiederhold (2018)^46,72^.
- Reasons for Returns and Environmental Implications: This section incorporated insights from Cullinane and Cullinane (2021) and Balaram et al. (2022)^73,74^.
- Attitudes Toward Second-Hand Clothing: Questions were derived from Tu et al. (2022), Mohammad et al. (2021), and de Aguiar Hugo et al. (2023)^75–77^.
- Purchase Considerations: Frameworks from Lu et al. (2022), Mandarić et al. (2022), and Diaz de Rada (1998) informed this Sects.^78–80^.

Demographic questions were included to examine variations in attitudes and behaviors by age, income, gender, and political engagement. All items were adapted to align with the specific objectives of this study.

The final questionnaire comprised 40 items, divided into three categories:

1. Demographic questions: capturing respondent characteristics;
2. Attitudinal questions: Addressing perceptions of fashion consumption and environmental issues; and.
3. Behavioral questions: Exploring purchasing patterns and environmental behaviors, with distinctions between new and secondhand clothing.

Several items were designed as matrix questions to facilitate the creation of calculated indices or variables, as detailed in subsequent sections. The full questionnaire is provided in Appendix A.

### Sampling strategy

The survey targeted a diverse sample of U.S.-based fashion consumers, defined as individuals aged 18 to 79 who had purchased clothing within the past five years. The sampling strategy employed stratified sampling to ensure representation across key demographic variables, including age, gender, and geographic location. Respondents were recruited from all 50 U.S. states, with age strata segmented into the following categories: 18–24, 25–34, 35–44, 45–54, 55–64, and 65+. Gender representation was balanced, with a minimum of 40% women and 40% men.

The survey was administered in two waves, yielding a final sample of 1,009 respondents. Approximately 70% of participants completed more than two-thirds of the survey. An early programming error in the survey platform led to elevated withdrawal rates during the first five days of data collection; however, this issue was subsequently corrected, after which dropout rates fell below 3%. Given the improved completion rate post-correction, no further adjustments to the sampling protocol were necessary. The final sample is intended to reflect the characteristics of U.S. fashion consumers rather than the general population.

### Pilot testing and validation procedures

Pilot testing was conducted in May 2024 with a sample of 60 voluntary participants to assess the reliability and usability of the survey instrument before full deployment. The pilot sample included respondents aged 20 to 78, with 67% between the ages of 25 and 55, and a balanced gender distribution. Participants were recruited from 37 U.S. states, representing all major geographic regions (East Coast, West Coast, South, and Midwest), using snowball sampling through the researchers’ professional and personal networks via email and WhatsApp groups. While these participants were not compensated, their feedback was instrumental in refining the survey’s design. The pilot revealed several issues with the initial version of the questionnaire, which included separate questions for each year from 2020 to 2024 regarding purchase frequency and spending. High dropout rates and increased failure on the alertness check indicated that this section was overly complex and burdensome. Verbal feedback from participants confirmed these concerns, citing the section’s length and difficulty. In response, the questionnaire was revised to consolidate the year-by-year questions into a single item assessing changes in purchasing behavior and spending over the 2020–2024 period. This adjustment reduced respondent fatigue and improved data quality while maintaining the integrity of the core variables.

### Data analysis

The survey’s matrix questions generated 13 calculated variables to assess various aspects of fashion consumption and sustainability. These variables are described below:

(i) **NewMoney_TOTAL**: Represents the total average monthly spending on new clothing from all types of businesses over the past year (Questions 3–6). Higher scores indicate greater expenditure.
(ii) **UsedMoney_TOTAL**: Represents the total average monthly spending on used clothing from all sources over the past year (Questions 9–12). Higher scores indicate greater expenditure.
(iii) **Used_ATTITUDE**: An index measuring attitudes toward used fashion products (Question 13). Higher scores signify more positive attitudes.
(iv) **Used_ACTIONS**: A sustainability index related to actions concerning used fashion products, particularly the practice of discarding clothes (Question 16). Higher scores indicate more sustainable behaviors.
(v) **Changes 2020–2024**: A sequence of items assessing whether respondents changed specific behaviors since 2020 (Question 14). An exploratory factor analysis of these items produced three indices:

- **Change.sust**: Measures shifts toward more sustainable fashion consumption, such as buying used clothes, selling used clothes, attending clothing swap events, and prioritizing sustainability in purchasing decisions. Higher scores indicate greater change toward sustainability.
- **Change.local**: Measures shifts toward purchasing locally produced products, such as clothes made in the USA and bought in physical stores. Higher scores indicate greater change toward local consumption.
- **Change.new**: Measures shifts toward fast-fashion consumption, such as buying new clothes online or purchasing from ultra-fast fashion chains. Higher scores indicate greater engagement with fast fashion.

(vi) **Returns**: A sustainability index assessing reasons for returning items, calculated as the weighted average of reasons provided, with sustainability values determined in consultation with a sustainable fashion expert (Question 19). Higher scores signify more sustainable return reasons.
(vii) **Purchase considerations**: This variable captures the importance of various considerations when purchasing clothing, rated on a 5-point Likert scale (Question 23).

An exploratory factor analysis yielded three indices:

- **Consider pragmatic**: Measures practical considerations, including fabric feel, functionality, sewing quality, fabric composition, and durability. Higher scores indicate more pragmatic purchasing behavior.
- **Consider sustain**: Measures sustainable considerations, such as environmental impact, maintenance requirements, fair labor practices, and products made in the USA. Higher scores indicate a stronger focus on sustainability.
- **Consider consume**: Measures consumerist considerations, such as price, anticipated usage frequency, appearance/design, and promotional discounts. Higher scores indicate more consumerist purchasing behavior.

(viii) **Knowledge**: An index measuring knowledge of sustainable fashion, based on the number of correct answers to a true/false question set (Question 26). Higher scores indicate greater knowledge (Range: 0–14).
(ix) **SustFashion_ATTIT**: An index measuring general attitudes toward sustainable fashion (Question 27). Lower scores indicate more positive attitudes.

In addition to individual variables and indices, three composite indices were calculated by combining multiple measures. Higher scores indicate more sustainable behaviors:

(x) **FastNew**: Measures the “speed” of new fashion consumption. A score of 5 indicates slower, more sustainable new fashion consumption, while a score of 1 indicates faster, less sustainable consumption. This index was calculated using reversed variables for NewNumber, NewFrequency, and Change.NewOnline, combined with NewMoney_TOTAL (Questions 1–6, Question 14).
(xi) **FastUsed**: Measures the “speed” of used fashion consumption. A score of 5 indicates slower, more sustainable used fashion consumption, while a score of 1 indicates faster, less sustainable consumption. This index was calculated using reversed variables for UsedNumber and UsedFrequency, combined with UsedMoney_TOTAL (Questions 7–12).
(xii) **FootPrint**: Represents the environmental footprint of respondents’ overall fashion purchasing patterns. This index was calculated as the mean score of several variables, including Used_ACTIONS, reversed variables for ReturnOnline.Frequency and ReturnOnline.Percent, Returns, DisposeTime, DisposeCondition, and a weighted mean of reasons for discarding clothing (Questions 16–21). Higher scores indicate a lower environmental footprint.

Exploratory Factor Analysis (EFA) with Varimax rotation was conducted and Cronbach Alpha reliability was calculated for each index. Detailed descriptive statistics of the indices are presented in Table 18 in Appendix B.

### Data analysis techniques

To examine the relationships between the study’s variables, multiple statistical approaches were employed:

1. **Correlation analysis**: We used Pearson’s r correlations to analyze relationships among the different indices, and Spearman’s Rho for correlations between quantity and frequency of clothing purchases (measured ordinally on a Likert scale).
2. **Bivariate analyses**: We tested Crosstabulations and associations between simple variables and indices by demographics using one-way ANOVA. For indices by demographics (e.g., the covariance of calculated variables with demographic variables), one-way ANOVA was performed across different levels of each demographic variable for each calculated index.

### Cluster analysis

To identify patterns within the survey data, k-means cluster analyses were conducted using variables and indices that demonstrated significant relationships with demographic characteristics in the bivariate analyses. The FastNew, **FastUsed**, andFootPrint indices were excluded from clustering to avoid endogeneity, as these indices incorporate other calculated variables.

The k-means clustering was performed in R using the *jamovi* GUI (The jamovi project, 2024, Version 2.5) and the snowCluster module (Seol, H., 2024, Version 7.4.1). Variables included in the clustering process were:

- **Simple variables**: NewNumber, NewFrequency, New.NotWorn, ReturnOnline.Frequency, DisposeCondition.
- **Indices**: NewMoney_TOTAL, UsedMoney_TOTAL, Used_ACTIONS, Change.Sust, Change.New, Knowledge, SustFashion_ATTIT.

A subsequent Principal Component Analysis (PCA) was performed on the identified clusters to explore their relationships with key demographic variables (e.g., age, gender, political engagement, employment status, student status, and retirement). The PCA explained 44.9% of the total variance in the data.

While PCA is excellent for dimensionality reduction and identifying variable structures, it does not directly segment respondents in the same way k-means does. Our sequential approach allowed us first to establish distinct consumer groups (via k-means) and then to use PCA as a complementary tool to visualize, interpret, and further describe these groups, particularly in relation to demographic characteristics. This approach allows for a clear distinction between identity markers (class, political affiliation, etc.) and behaviors and attitudes, and it prevents endogeneity when directly examining the relationships among variables from the two groups.

This multi-method analytical approach provided a comprehensive understanding of the relationships among consumer behavior, fashion consumption, and sustainability outcomes.

### Ethical approval and participant consent

This study was approved by the Yale University Ethics Committee (IRB Protocol ID: 2000037433) on March 11, 2024, following an initial review. The title of the study is “Examining Fashion Consumption Patterns, from an Environmental Perspective, in the USA.” All experiments and procedures were conducted in accordance with relevant guidelines and regulations outlined by the committee. Informed consent was obtained from all participants prior to their inclusion in the study. The consent process was documented through a signed consent form (Consent Form: Fashion.docx). Supporting documents submitted as part of the IRB approval included recruitment materials (Questionnaire A and B), the study protocol, and a Faculty Advisor Agreement.

## Results

### Sample demographics

A total of 1,009 individuals from all states in the United States participated in the survey. Among these participants, 46% completed the entire questionnaire, while 70% answered more than two-thirds of the questions. The demographic profile showed that 45.7% were aged 25–44, and 55.7% of participants were women. Respondents holding an academic degree accounted for 72% of the sample, and 77.3% reported annual household incomes below $100,000. Employment data indicated that 58% were employed either part-time or full-time. Additionally, 57% were single, and 22% reported active political engagement. For a detailed demographic breakdown, refer to Tables 1, 2, 3, 4, 5, 6 and 7 in Appendix B.

### Primary and secondary market statistics

Across the sample, participants demonstrated significant engagement with both primary (new clothing) and secondary (used clothing) markets. Analysis revealed that 69.4% of respondents purchased secondhand clothing at least once, while 85% reported purchasing 10 or fewer new items per month, and 87.3% purchased 10 or fewer used items per month.

Between 2020 and 2024, the data showed a 38% increase in online purchases of new clothing, a 24.6% rise in ultra-fast fashion purchases from platforms such as SHEIN and Temu, and a 40.8% increase in buying new clothes at lower prices than they have paid before. In contrast, sustainable practices, including clothes-swapping and selling used clothing, declined during the same period (see Tables 8, 9 and 10 in Appendix B). These shifts highlight a move toward less sustainable practices over time, despite the expanding secondary market, supporting the hypothesis that secondary market growth may facilitate rather than reduce overall consumption.

### Relationship between primary and secondary markets

The analysis revealed a significant positive correlation (*r* = 0.58, *p* < 0.01) between spending in primary and secondary markets. This finding indicates that consumers who purchase more new clothing also tend to buy more used clothing, suggesting that the secondary market complements, rather than displaces, primary market consumption. Additional correlations between purchase quantities (*r* = 0.317, *p* < 0.01) and purchase frequencies (*r* = 0.256, *p* < 0.01) further underscore the complementary nature of the two markets, indicating that increased activity in one domain is associated with increased activity in the other.

Younger respondents showed higher purchasing volumes and frequencies in both markets (ρ = 0.205, *p* < 0.01), highlighting a clear relationship between age and purchasing behavior. High-spending individuals in either market were also more likely to exhibit frequent purchasing behavior. Additionally, participants in the secondary market reported significantly higher purchase frequencies (F = 15.3, *p* < 0.01) as compared to non-participants. These results support H2, indicating that participation in the secondary market does not reduce overall consumption but instead sustains or amplifies it.

### Consumption and disposal behaviors

Approximately 19% of respondents reported discarding clothing specifically to make room for new purchases, while 43% discarded items because they were “no longer pleasing,” out of fashion, or due to a desire for renewal (see Table 11 in Appendix B). Supporting this, 25% of respondents discarded clothing that was still in good condition (see Table 12 in Appendix B). Notably, 40% of respondents owned clothing they had never worn, and 37.9% disposed of items within a year of purchase, including 14.2% within just one month. Respondents also reported high rates of returning purchased clothing, particularly in the context of online shopping (see Tables 12, 13 and 14 in Appendix B). This supports H9, as higher purchasing volumes and frequencies are associated with more frequent returns.

The most common method of clothing disposal was donation to secondhand charity stores, reported by 45.3% of respondents. While this form of disposal is widely practiced, more sustainable forms of reuse, such as selling used clothing, remain less common; 41.7% of respondents indicated they had never sold any garments. These patterns reflect a reliance on familiar and convenient disposal channels. As a result, consumption and disposal behaviors may support the continued activity of both primary and secondary markets without necessarily reducing overall material throughput. This supports H1, as higher purchasing activity in both markets is associated with greater textile disposal volumes. This pattern also aligns with rebound-effect dynamics, whereby perceived environmental benefits from buying used clothing may encourage greater overall acquisition and faster turnover, ultimately undermining sustainability gains.

Between 2020 and 2024, 37.2% of respondents reported increasing their clothing donations; however, this behavior coincided with a 38% rise in new clothing purchases, suggesting that donation practices occurred alongside, rather than in place of, continued acquisition. At the same time, sustainable maintenance practices remained low, with only 18.9% of respondents reporting attempts to repair their clothing (see Table 15 in Appendix B).

### Behavioral insights

**Fig. 1:**
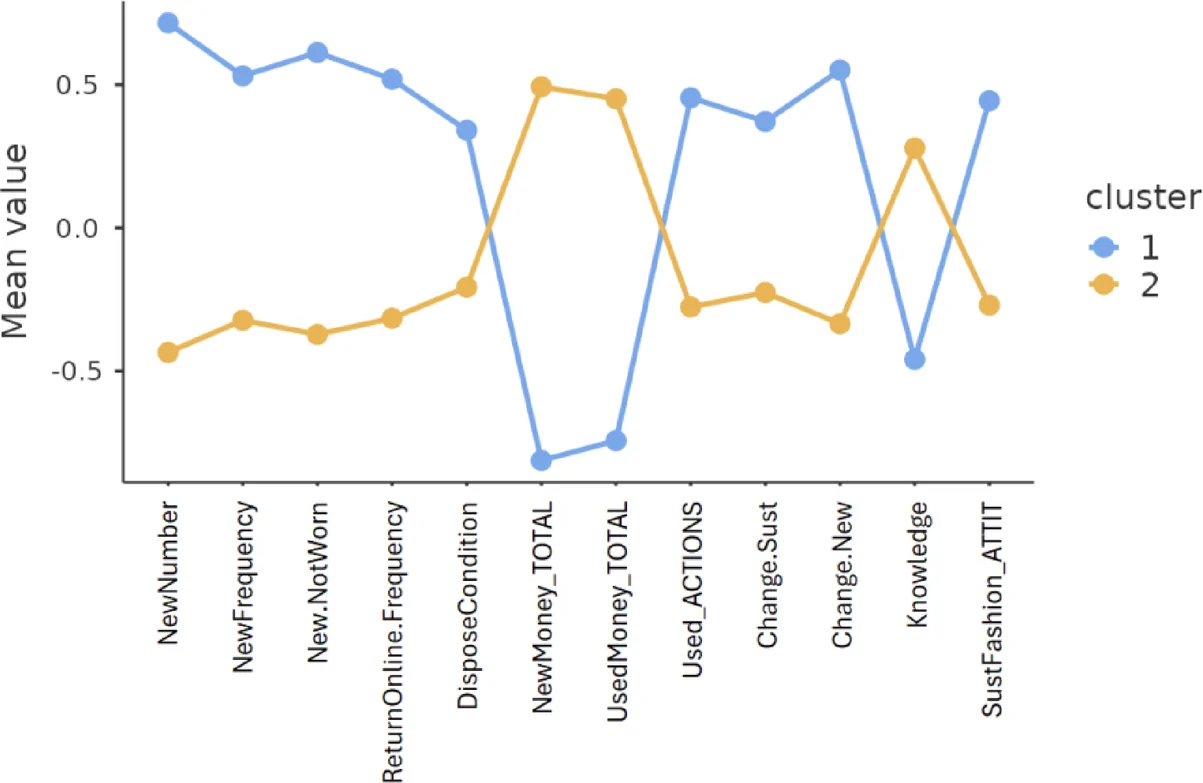
Clusters and Variables.

The analysis identified two distinct behavioral groups based on key variables, including purchase frequency, spending patterns, disposal behaviors, and environmental awareness (see Fig. 1).

**Cluster 1**, comprising 59% of respondents, was characterized by high consumption levels in both new and secondhand markets. This group demonstrated frequent returns, shorter garment retention periods, and an increase in the purchase of secondhand clothing since 2020. Additionally, Cluster 1 respondents exhibited lesser knowledge about sustainability and less favorable attitudes toward sustainable fashion. Their purchasing patterns suggest that secondhand consumption may mimic the behaviors associated with fast fashion.

In contrast, **Cluster 2**, which included 41% of respondents, showed lower consumption levels, higher spending per item, and longer garment retention periods. While this group displayed greater environmental awareness, they were less likely to adopt sustainable disposal methods such as reselling used clothing. This disconnect highlights a gap between these respondents’ sustainability knowledge and their actual practices.

The relationship between employment status and consumption patterns remains significant even when controlling for income (F = 14.1, *p* < 0.01), underscoring the role of social and occupational contexts in shaping behaviors.

**Fig. 2:**
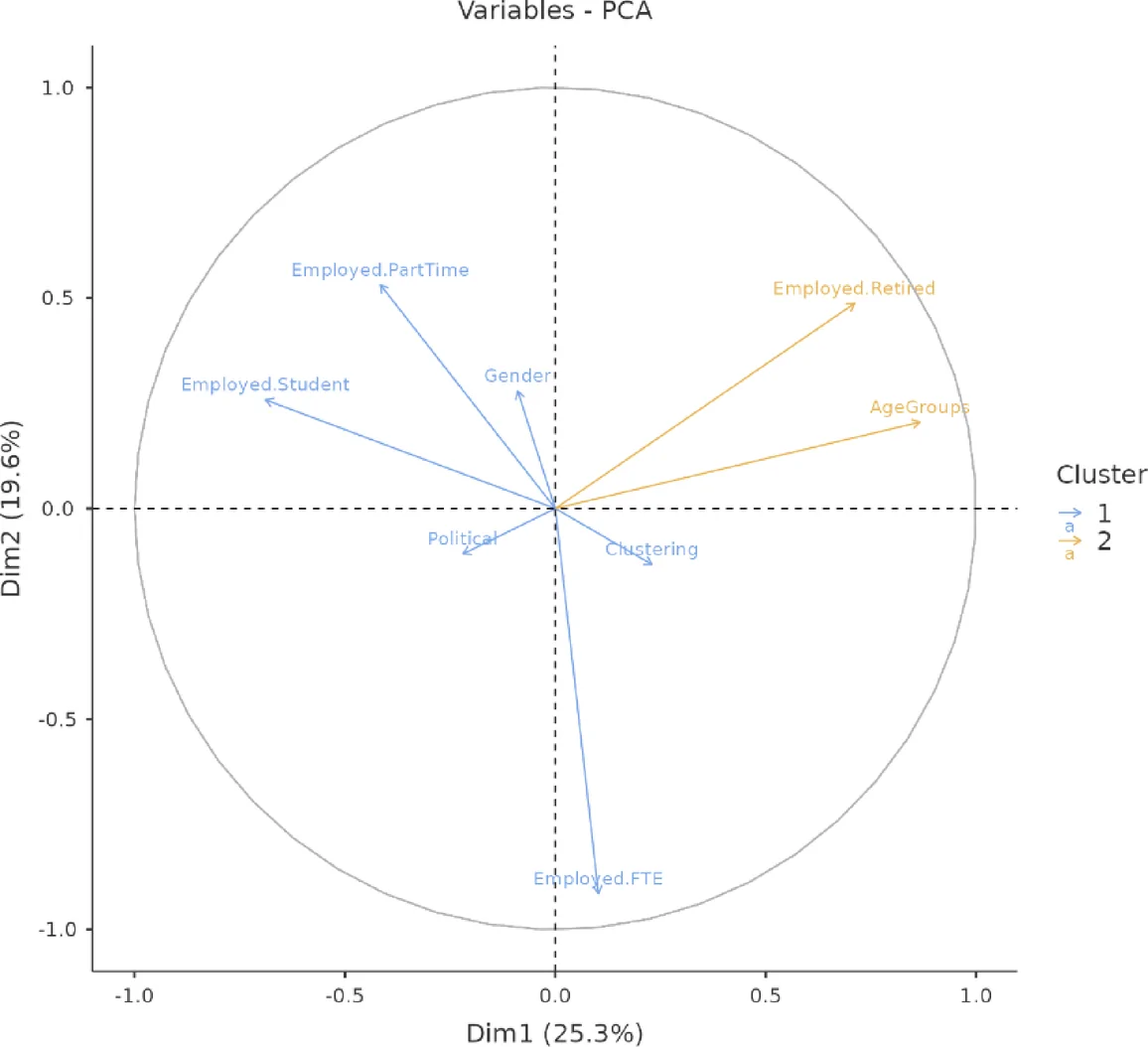
Principal Component Analysis Chart.

Principal Component Analysis identified a strong association between Cluster 1 and students or employed individuals, whereas Cluster 2 was linked to older and retired individuals (see Fig. 2).

An analysis of the two clusters reveals distinct demographic profiles (full statistics are provided in Table 16 in Appendix B). While both groups are close to gender-balanced, Cluster 1 is primarily composed of individuals with lower socioeconomic status: 50% report annual household incomes below $50,000, and 60% have less than a college degree. A majority of Cluster 1 members (60%) are single. Cluster 2, by contrast, is more educated and financially stable, with only 39% earning below $50,000 and 40% lacking a college degree. Cluster 2 is also older on average, with individuals over 55 comprising 33% of its members, compared to just 20% in Cluster 1.

### Demographic insights

Our exploration of the third research question—*how do demographic characteristics*,* including age*,* gender*,* income*,* employment status*,* and political engagement*,* relate to consumption patterns across markets?* —yielded the following insights:

#### Age

**Fig. 3:**
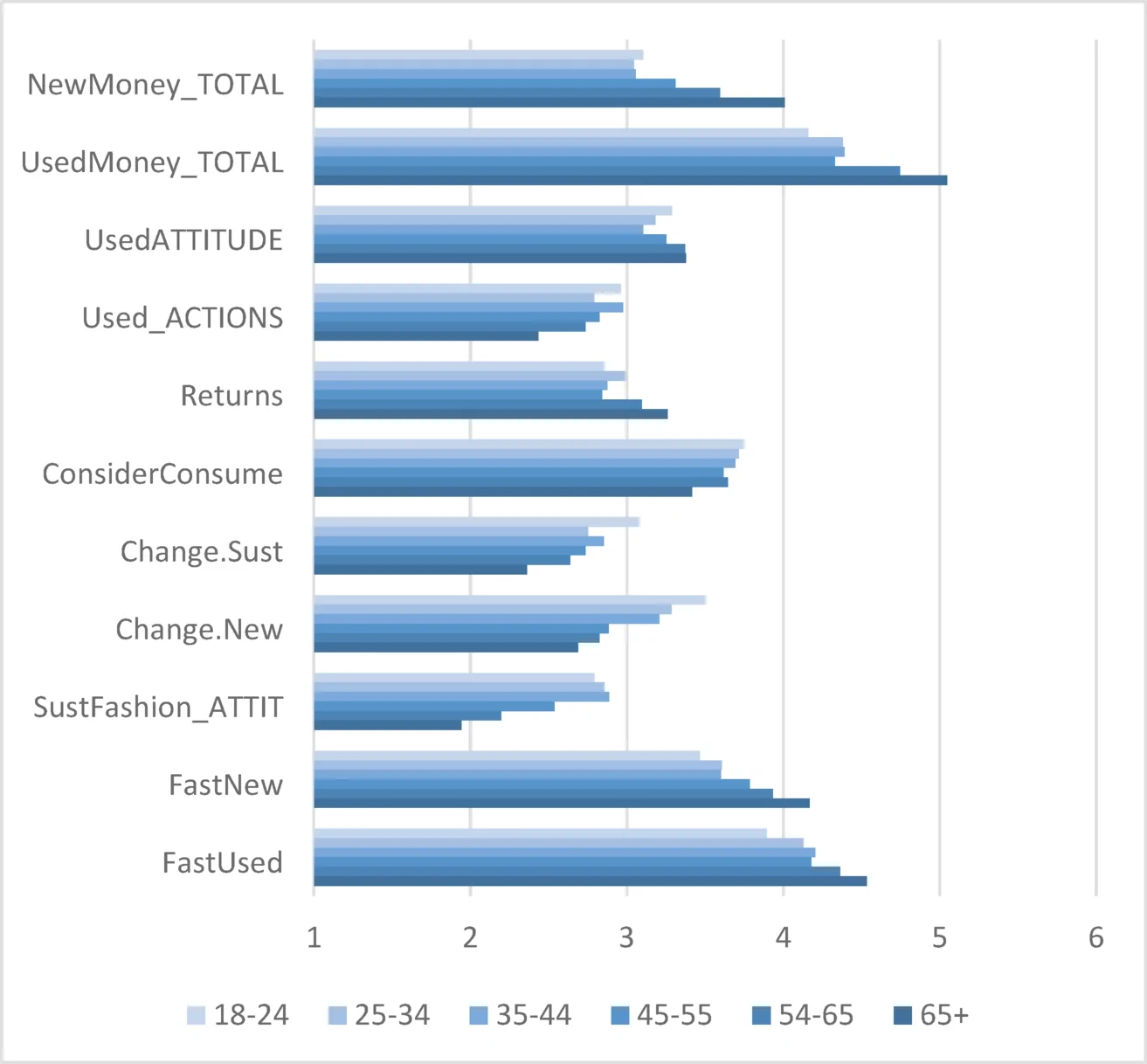
Indices calculated by age.

Younger respondents reported higher purchasing volumes and frequencies in both primary and secondary fashion markets (*r* = 0.205, *p* < 0.01). This offers limited support for H3, as younger consumers reported higher purchasing volumes and frequencies for new clothing than older consumers; however, the correlation is weak and should be interpreted with caution until tested further. The popularity of secondhand clothing was especially pronounced among younger individuals, with 79% of respondents aged 18–24 purchasing secondhand clothing, as compared to 57% of those aged 65 and older (see Fig. 3). This supports H4, as younger respondents also have the highest rates of secondhand purchases, with participation declining with age. This finding also aligns with data showing that two out of five items in Gen Z wardrobes are secondhand^6^.

Younger consumers also reported owning more unworn items, reflecting the extension of fast-fashion behaviors into the secondary market. These consumers engaged in frequent, lower-cost purchases, which further highlights the dual engagement of younger individuals across both primary and secondary markets. This behavior underscores the potential for the rebound effect, where participation in the secondary market, perceived as a sustainable practice, may inadvertently drive increased consumption rather than mitigate it.

In contrast, older individuals spent more per item, purchased fewer items, and retained clothing for longer periods. These patterns suggest that secondhand consumerism among younger individuals is primarily driven by affordability, whereas older consumers prioritize durability and longevity in their purchasing decisions.

**Fig. 4:**
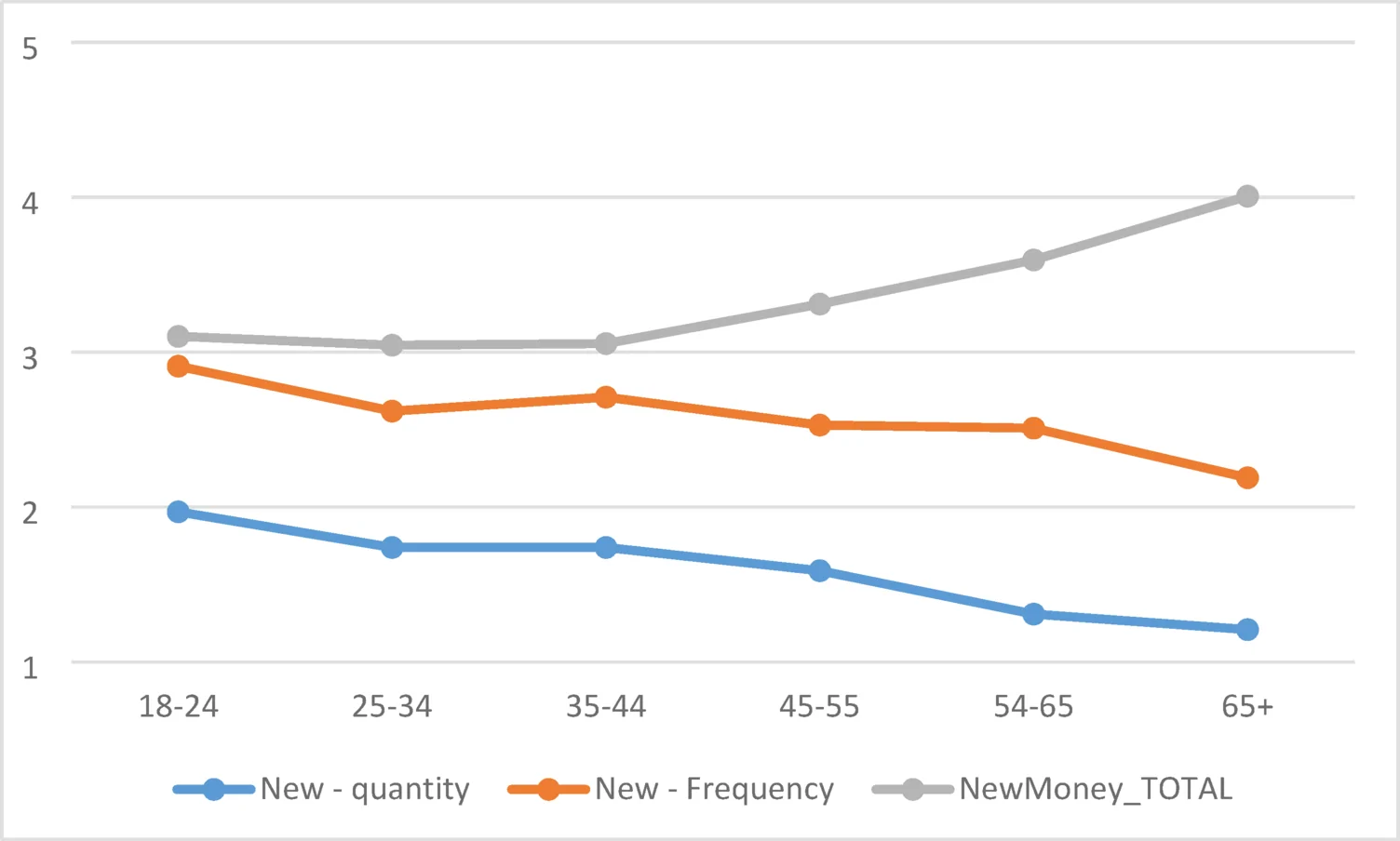
Frequency, quantity, and total expenditure by age group.

Figure 4 illustrates the relationship between age and purchasing behaviors across markets. A generalized linear model tested this relationship, yielding a Likelihood Ratio Chi-Square of 92.04 (df = 11, *p* < 0.001). The analysis revealed a significant main effect of FastUsed, indicating that individuals who purchase more new clothing tend to score lower on sustainable secondhand purchasing behaviors. Age did not significantly influence this relationship.

#### Income

**Fig. 5:**
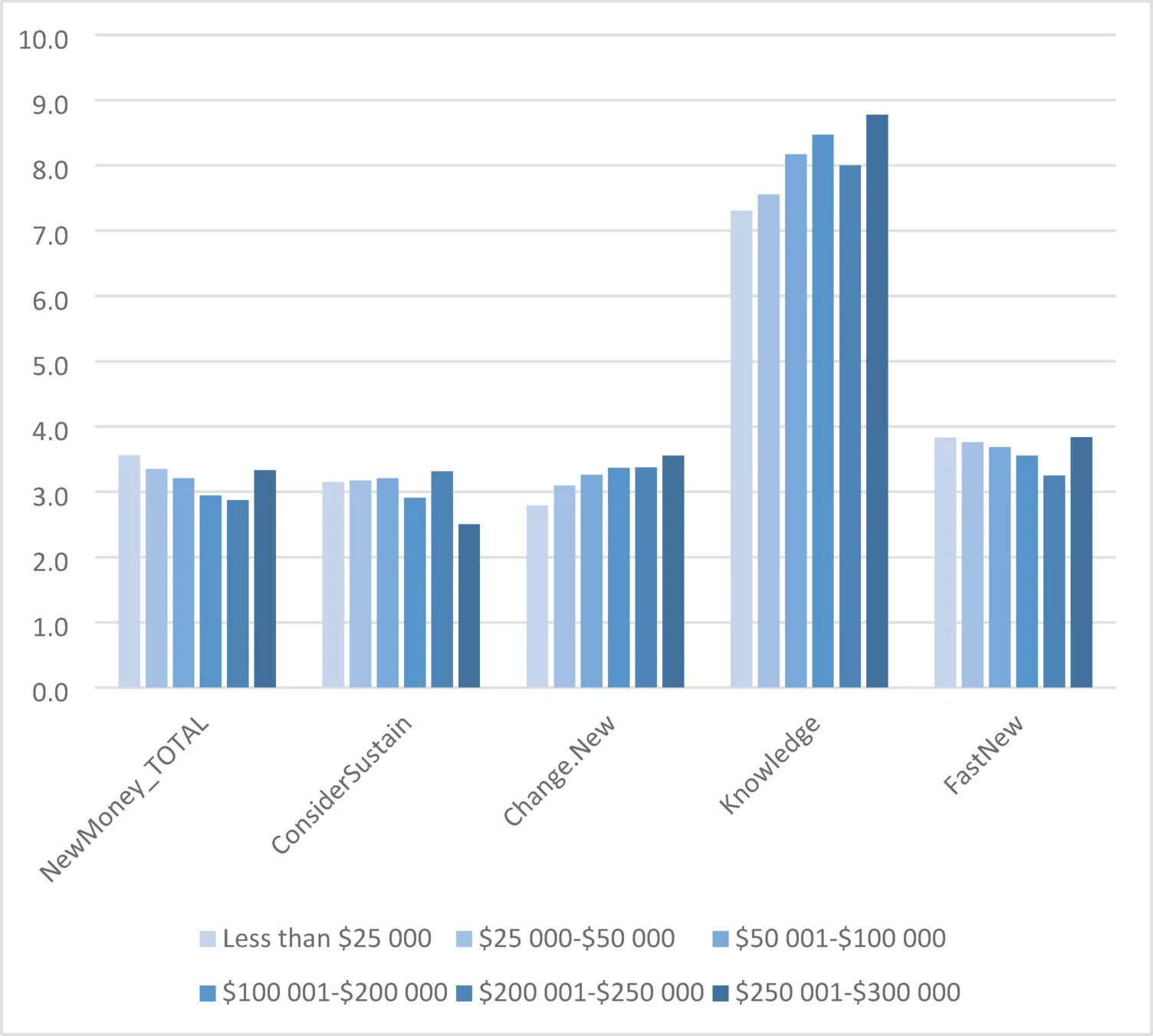
Indices calculated by income.

The analysis revealed that higher-income respondents spent more on both new and used clothing (F = 14.1, *p* < 0.01). This group also exhibited shorter garment retention periods and a higher likelihood of discarding items in good condition, indicating a greater overall level of consumption (see Fig. 5). Although higher-income individuals demonstrated greater environmental knowledge, their consumption behaviors were less sustainable, as reflected in higher return rates and a tendency toward conspicuous consumption. If so, H7 is not supported, as higher education and knowledge levels did not correspond to lower new clothing consumption. These spending patterns reinforce the complementary relationship between the secondary and primary markets, rather than a substitutional one.

#### Gender

**Fig. 6:**
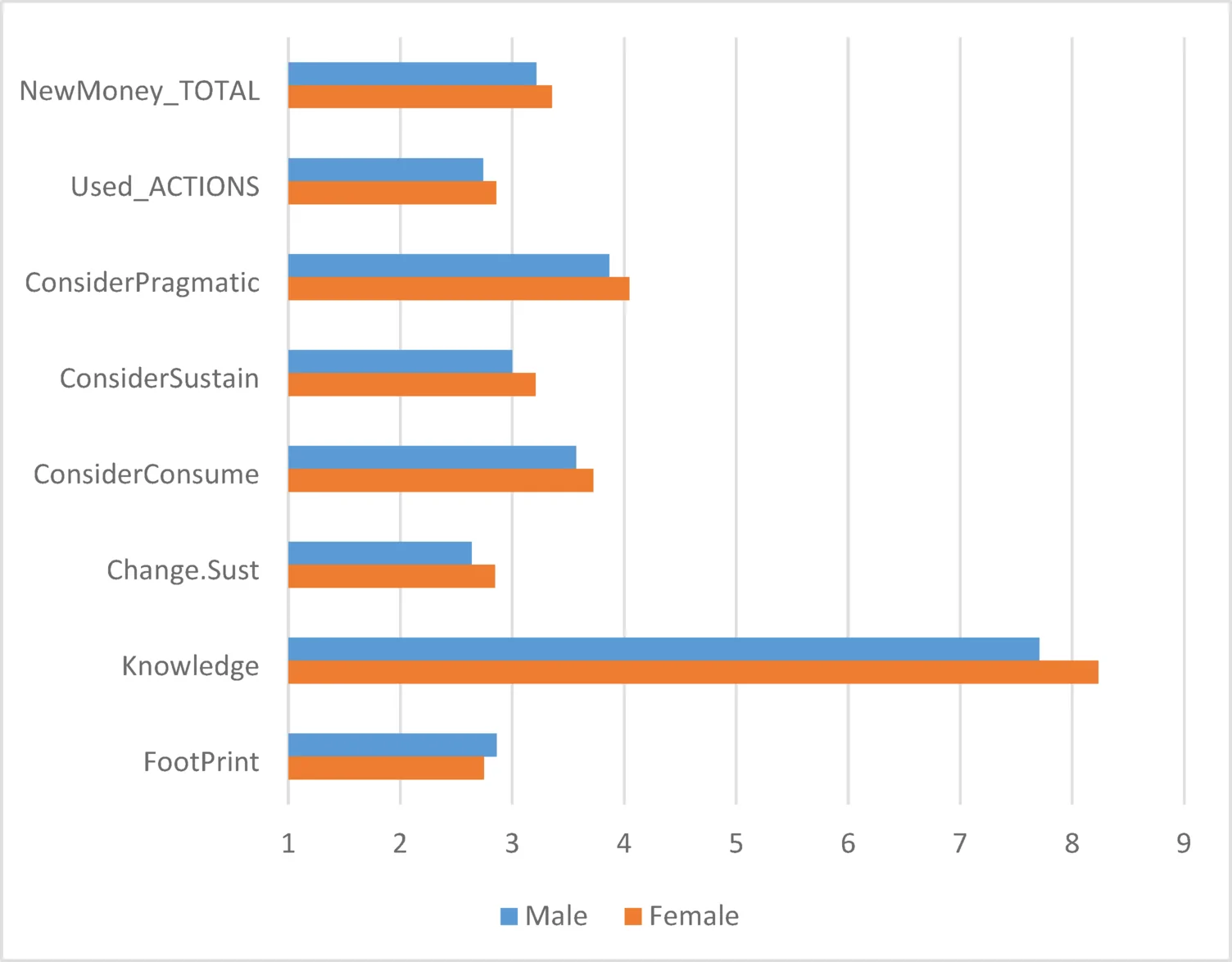
Indices Calculated by Gender.

Women demonstrated greater engagement with both primary and secondary markets, purchasing more frequently and in higher volumes than men. This supports H5. Women showed more positive attitudes toward sustainability, greater knowledge of the environmental impact of the fashion industry, and higher consideration for sustainability in purchasing decisions, and they were also more likely than men to engage in sustainable disposal practices, such as repurposing clothing. This supports H6. Nevertheless, women’s overall consumption patterns resulted in a larger environmental footprint than men’s, as reflected in footprint indices (see Fig. 6), suggestive evidence of the rebound effect.

#### Employment status

Students were the most likely to purchase secondhand clothing, with 84% reporting such purchases compared to 69% of the total sample. In contrast, retired individuals demonstrated more sustainable consumption patterns, characterized by lower purchase volumes and longer garment retention periods, despite having less favorable attitudes toward sustainable fashion.

While students exhibited higher levels of knowledge about the environmental impact of the fashion industry, they scored the lowest (2.9) on sustainability considerations when making purchasing decisions.

#### Political Involvement

**Fig. 7:**
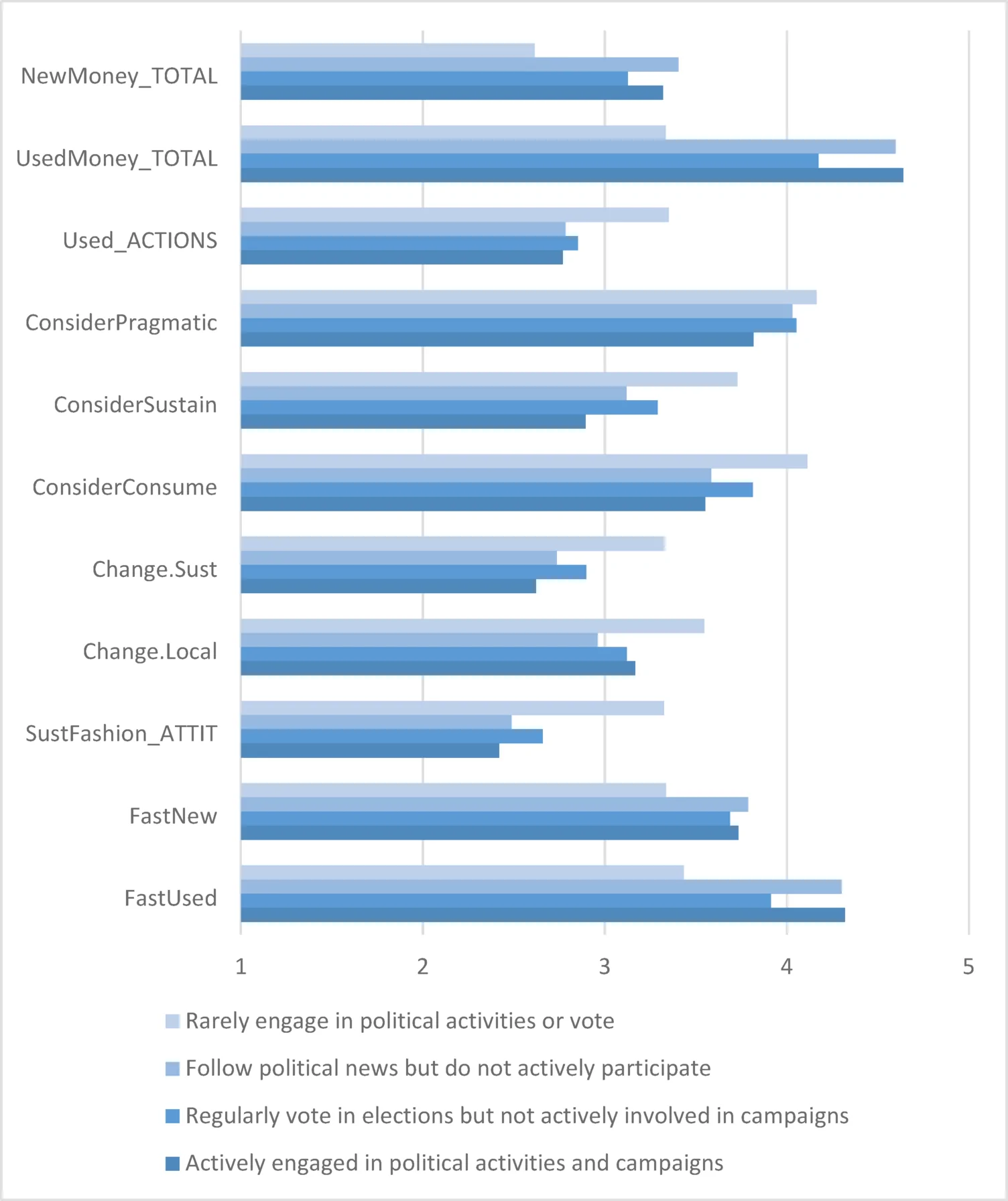
Indices According to Political Involvement.

The relationship between political activism and fashion consumption is mixed. Politically engaged respondents spent less money on new fashion overall; however, on the *FastNew* index, which incorporates both quantity and frequency of purchases, the least politically active scored lowest (see Fig. 7). Less politically active respondents also scored higher on several sustainable fashion indices, including attitudes toward sustainable fashion and certain actions involving used garments. Yet this same group ranked highest on consumerist purchasing considerations and lowest on the *FastUsed* index, indicating greater quantity and frequency of used clothing purchases. They were also more likely to own unworn new garments; report higher rates and frequencies of online returns; and dispose of clothing while it was still in good condition. H8 is partially supported —politically engaged respondents spend more per secondhand item but purchase them less frequently than less engaged individuals.

This complex pattern suggests that lower political engagement may coincide with both resource-driven limitations on new fashion purchases and behaviors that remain environmentally unsustainable. In some cases, these patterns may reflect moral licensing, where engagement in perceived sustainable practices, such as buying used clothing, coexists with high overall consumption.

### Knowledge and sustainability

Contrary to expectations, greater knowledge about the environmental impacts of fashion was negatively correlated with sustainable purchasing and disposal behaviors. For example, sustainability knowledge showed a small but statistically significant negative correlation with sustainable actions (*r* = − 0.095, *p* < 0.05). While the effect size is modest and should be interpreted with caution, the direction of the relationship is nonetheless noteworthy. Respondents with higher sustainability knowledge often reported higher overall consumption levels across both markets, suggesting a possible disconnect between awareness and action (for full results, see Table 17 in Appendix B). Therefore, H10 is not supported, as greater sustainability knowledge was linked to less sustainable purchasing and disposal behaviors. This is consistent with moral licensing dynamics, in which perceived ethical behaviors, such as purchasing secondhand clothing, serve to justify continued or increased consumption.

## Discussion

### Core finding

The relationship between primary and secondary fashion markets reveals a complex dynamic that challenges conventional assumptions about sustainable consumption. While secondary markets are often celebrated for extending garment lifespans and reducing waste, our findings highlight a rebound effect that warrants a critical re-evaluation of the belief that secondhand consumption inherently mitigates the environmental impact of fashion. Instead of displacing consumption in the primary market, the growth of secondary markets appears to complement it. This suggests that both markets collectively sustain high levels of overall consumption, undermining the environmental benefits typically attributed to secondhand practices.

#### Parallel growth of consumption in both markets

A key insight from our analysis is the positive correlation between expenditures in primary (new) and secondary (used) markets, indicating that consumers who spend more in one market also tend to spend more in the other. This finding challenges the assumption that the secondary market serves as a direct alternative to the primary market. Instead, engagement with secondhand fashion appears to reinforce, rather than replace, patterns of high consumption. Additional correlations between purchase quantities and frequencies support the notion that engagement in the secondhand market does not necessarily replace new purchases but may instead accompany or even reinforce them, suggesting a complementary rather than substitutive relationship between the two.

The strongest interpretation of these findings reveals a potential paradox: rather than curbing overconsumption, secondary markets may inadvertently encourage unsustainable purchasing patterns. By facilitating both the acquisition and the disposal of garments, these markets can contribute to a self-reinforcing cycle of consumption. This is reflected in disposal behaviors that appear driven more by the desire for novelty, space, or trend alignment than by actual necessity. Many respondents reported discarding garments to make room for new purchases or due to fashion obsolescence, even when the items remained in good condition. These patterns suggest that secondhand consumption may often function within the logic of fast fashion rather than offer a true counterpoint to it. These dynamics align with the “sustainability bias” described by Colasante and D’Adamo (2021), where secondary markets project environmental benefits but coexist with continued overproduction, limiting their overall impact^46^. Similarly, Persson and Hinton (2023) argue that without regulatory frameworks to support a transition to a more circular fashion economy, secondary markets risk perpetuating existing production and consumption patterns rather than transforming them^5^. This pattern is consistent with the concept of *moral licensing*, i.e., the sense that engaging in seemingly ethical behaviors such as buying secondhand justifies subsequent unsustainable choices^83^. Additionally, the observed outcomes reflect a classic *rebound effect*, where the perceived reduction in environmental harm from one action leads to offsetting behaviors that undermine the net benefit^10^.

#### Conspicuous wasteful consumption patterns

Our cluster analysis highlights the prevalence of conspicuous consumption behaviors among a majority group (59%) of respondents. Members of this group frequently purchase clothing and retain garments for shorter periods, with 37.9% disposing of items within a year, and 14.2% discarding them within just one month. Additionally, this group reports a high rate of returns, particularly for online purchases. These behaviors reflect the rapid-turnover feature of fast fashion, which is facilitated by the secondary market.

While this group engages in seemingly sustainable disposal practices, such as donating garments or passing them on to family and friends, these practices do little to mitigate the broader environmental impact of rapid consumption cycles. Rather than reducing overall acquisition, such practices may offer a sense of entitlement or relief, allowing individuals to continue purchasing at a high rate while feeling that they are acting responsibly, consistent with the theory of moral licensing^83,84^.

#### Consumption patterns and demographic factors

Our findings reveal nuanced relationships between consumption patterns and demographic factors. While higher levels of education correlate with slightly improved environmental footprints, higher-income households have mixed sustainability profiles. On one hand, these households exhibit increased knowledge about sustainable fashion practices, aligning with prior research on environmental awareness^85^. On the other hand, they report higher rates of returns and greater overall spending, reinforcing unsustainable consumption patterns. This dynamic suggests that while education positively influences attitudes, income-driven behaviors often undermine sustainability goals. Economic incentives, such as affordability in secondary markets, may exacerbate consumption patterns when combined with cultural norms that prioritize acquisition over sufficiency, regardless of income.

These dynamics invite comparison with prior work on sustainability behavior and consumption psychology. Our findings both support and extend the insights of Olson (2022) and Hall et al. (2018). Olson demonstrated that sustainability-focused marketing in the fast fashion sector may produce rebound effects, encouraging additional consumption by creating a perception of ethical behavior. We observe a similar behavioral pattern in the resale context: individuals who actively participate in secondhand markets tend to consume more overall, suggesting that resale can justify or enable continued overconsumption. While Olson relied on experimental designs focused on short-term reactions to sustainability messaging, however, our findings draw from reported real-world behavior across time and market channels. This distinction implies that rebound effects in secondhand fashion may not only stem from messaging, it may also be structurally embedded in resale platforms. Specifically, low prices reduce the economic barrier to overconsumption; fast inventory turnover fosters a sense of urgency and novelty; and the belief that used items are inherently low-impact may lead consumers to treat them as disposable. When these features intersect with broader cultural norms that valorize trend responsiveness and wardrobe turnover, secondhand consumption risks reproducing the unsustainable behaviors it purports to challenge.

Likewise, our findings resonate with Hall et al.’s observation that pro-environmental attitudes do not reliably predict sustainable behavior. In our sample, greater sustainability knowledge was only weakly or negatively correlated with sustainable purchasing and disposal practices. Despite increased awareness, many respondents continued to purchase and discard clothing at high rates. This supports the idea of moral licensing, whereby prior ethical actions (e.g., buying used clothing) serve to justify less sustainable choices thereafter. Hall et al. conceptualize sustainability behavior as expressive or identity-driven, often decoupled from actual impact. Our results reinforce this claim but also extend it: by analyzing observed behavior (rather than short-term intentions), we show how knowledge, platform design, and weak systemic constraints contribute to this disjunction.

Demographic patterns further illuminate the mismatch between sustainability attitudes and actual behavior. Younger consumers (ages 18–24) reported higher purchasing volumes in both new and used markets (*r* = 0.205, *p* < 0.01), with 79% buying secondhand—compared to 57% among those over 65. Yet this group also reported more unworn items and frequent low-cost purchases, indicating that fast fashion behaviors persist despite secondhand engagement. Similarly, students, who showed high environmental knowledge and the highest secondhand purchase rate (84%), scored lowest on sustainability-related considerations when making purchases.

These findings support the presence of rebound and moral licensing effects: consumers may use their knowledge or secondhand participation as justification for continued overconsumption. In contrast, older and retired individuals reported more sustainable behaviors, such as buying fewer items, spending more per item, and keeping garments longer. Yet they did not express stronger pro-sustainability attitudes. This divergence underscores that sustainable behavior may be more closely shaped by structural and life-stage factors such as employment status, income stability, and exposure to market dynamics, rather than by values or knowledge alone.

Gender analysis also reveals complex patterns, again reflecting a disconnect between consumers’ sustainability knowledge and purchasing behavior. Women exhibit higher engagement with both primary and secondary markets and report stronger sustainability attitudes and knowledge. Yet, their higher purchase frequency offsets the environmental benefits of more frequent participation in sustainable disposal methods, resulting in greater overall environmental impacts.

Employment status provides further insights into consumption patterns. Cluster analysis revealed that students and employed individuals (Cluster 1) exhibit higher consumption levels across both markets as compared to retired and older individuals (Cluster 2). This finding suggests that market participation is influenced more by lifestyle and social factors than by economic necessity or environmental concerns.

These demographic patterns collectively challenge traditional assumptions about sustainability awareness and behavior. Younger, employed, and more educated consumers—groups typically associated with greater environmental consciousness^86^—exhibit consumption patterns that undermine sustainability goals. These consumers’ high engagement with both primary and secondary markets reflects a consumption model that sustains, rather than reduces, total consumption. The presence of a rebound effect is evident in the behaviors of Cluster 1, characterized by frequent returns, shorter retention periods, and high purchasing levels in both markets. These traits mirror fast-fashion consumption patterns and underscore how secondhand platforms may inadvertently fuel overconsumption instead of mitigating it.

While certain demographic patterns we observed, such as younger, female consumers purchasing more secondhand clothing than other groups, are consistent with established trends in fashion behavior, this study contributes to the literature by uncovering the psychological dynamics that accompany these behaviors. Specifically, the data reflect a high level of concern for sustainability among these groups, alongside their elevated consumption rates, suggesting a complex interplay between pro-environmental attitudes and purchasing behavior. This paradox aligns with existing theories of moral licensing and rebound effects, indicating that sustainability-oriented intentions may inadvertently justify or encourage higher levels of overall consumption^87,88^. Rather than interpret these patterns as mere contradictions or “hypocrisy,” our analysis positions them within a broader behavioral framework that helps explain how well-intentioned consumers may unintentionally undermine their own environmental goals. In doing so, the study moves beyond documenting consumer trends to theorizing the motivations and justifications behind them, an essential step for designing interventions that target not just behaviors but the cognitive processes underlying them.

### Mechanisms driving overconsumption

Our findings are best interpreted through two complementary theoretical frameworks: the rebound effect and moral licensing. These frameworks illuminate how seemingly sustainable behaviors, such as participation in the secondary fashion market, can inadvertently perpetuate or even amplify unsustainable consumption patterns, ultimately undermining environmental goals.

The rebound effect, rooted in environmental economics, occurs when perceived environmental or economic benefits lead to increased consumption, offsetting initial sustainability gains^11–13^. Rebound effects arise when efficiency improvements reduce the unit price of a good or service, prompting greater demand^89^. This dynamic is evident in our study, where secondary market participants exhibited higher purchase volumes in both primary and secondary markets (*r* = 0.58, *p* < 0.01)^84^.

The moral licensing framework provides psychological insights into this paradox. Moral licensing occurs when individuals justify unsustainable actions by referencing prior virtuous behaviors, effectively “offsetting” the guilt of subsequent unsustainable choices^90,91^. Our findings reflect this dynamic: although 45% of respondents reported donating clothing to secondhand stores, these ostensibly sustainable actions coexisted with sustained high consumption in both primary and secondary markets^89^.

This disconnect between intentions and behaviors exemplifies the “ethical purchasing gap”^85^. While consumers increasingly express concern for environmental issues, this concern rarely translates into sustainable actions. For instance, although 37.2% of respondents reported increasing their clothing donations between 2020 and 2024, this behavior coincided with a 38% rise in new clothing purchases. These findings illustrate how secondary markets often enable overconsumption rather than curb it.

Previous studies reinforce these findings. Geng et al. (2016) observed that consumers who chose green products reported reduced intentions to engage in subsequent pro-environmental behaviors^92^. Similarly, Khan and Dhar (2006) demonstrated that performing virtuous acts such as donating often led to indulgent behaviors^91^. Friedrichsmeier and Matthies (2015) further showed that sacrifices in one area often resulted in indulgence in another^93^. This dynamic aligns with our findings, which reveal that Cluster 1 exhibited high consumption levels across both markets despite expressing greater environmental awareness.

This highlights the limitations of consumer-driven solutions in addressing the structural challenges of the fashion industry. Without tackling the root causes of overproduction and the normalization of overconsumption, secondary markets risk reinforcing the very patterns they aim to disrupt. The following section presents actionable policy recommendations derived from our findings, aimed at addressing these systemic drivers of overproduction and overconsumption.

### Policy and practice implications

#### Conclusion and policy implications

This study provides empirical support for a more cautious approach to policies that promote secondhand markets as inherently sustainable. Our findings reveal that secondary market participation often coexists with continued high levels of new clothing consumption. This challenges the assumption that secondhand markets displace primary market demand or significantly reduce textile waste.

Our results underscore the need for policymakers to refrain from treating secondary markets as categorically benign. Regulatory attention must extend to resale platforms, donation-based enterprises, and peer-to-peer markets. Prior research suggests that key interventions may include:

- Requiring resale platforms to disclose sustainability metrics, including unsold inventory disposal rates, shipping-related emissions, and marketing practices that incentivize overconsumption^14,57,95^;
- Clarifying the environmental impact of donation-based organizations, particularly with respect to incineration or landfill rates of low-quality items that cannot be resold^46^.
- Reducing reliance on awareness-based campaigns, as our data show that knowledge about the environmental and social harms associated with the apparel industry does not consistently lead to more sustainable consumption patterns, echoing other findings on the limits of education in changing behavior^96,97^.

Promoting certification systems to verify and communicate the environmental impacts of fashion brands and resale platforms would strengthen accountability and support evidence-based policymaking. While we acknowledge that information provision alone does not reliably shift consumer behavior, a conclusion supported by both our findings and prior research^29,94–97^transparency remains vital for regulatory purposes. Our recommendation is not aimed at increasing consumer awareness per se, but rather at equipping policymakers with standardized, reliable data to assess the environmental performance of both primary and secondary market actors. Such transparency is a prerequisite for developing effective interventions that go beyond individual education and address systemic market failures^98–100^. Mandating public reporting on sustainability metrics, including unsold inventory management, shipping emissions, and marketing strategies, would allow regulators to monitor whether resale platforms are reproducing fast fashion dynamics under a sustainable veneer.

At the same time, our findings, like those of Olson and Hall, underscore the need to recognize the emotional and hedonic dimensions of consumption. Many consumers derive pleasure, identity affirmation, or a sense of novelty from shopping, regardless of whether they purchase new or used items. This behavioral reality complicates demand-side interventions and highlights the importance of regulating supply-side drivers of overconsumption. Thus, increasing transparency is not about assuming that better-informed consumers will behave sustainably, but about creating the regulatory foundation for reshaping the market itself, reducing incentives for overproduction, tempering exploitative marketing, and aligning the resale economy with broader sustainability goals.

Our findings also challenge the assumption that pro-sustainability attitudes alone are sufficient to drive meaningful reductions in consumption. Accordingly, behavior-oriented interventions such as environmental cost accounting, taxes on non-durable garments, or default settings that prioritize longer-lasting goods, deserve further exploration^96^.

Policy strategies must move beyond promoting secondary markets in isolation and address the broader systems that drive overconsumption. These may include stricter regulation of secondary market actors and efforts to encourage a cultural shift from acquisition to sufficiency, that is, promoting reductions in total consumption rather than merely shifting the source of purchases^101^.

#### Implications for business and practice

Our findings suggest that businesses in the resale sector should align operational practices with demonstrable sustainability outcomes rather than rely on circularity rhetoric. Specifically:

- Resale platforms should adopt transparent business models that communicate product sourcing, reuse practices, and environmental trade-offs to consumers^95^.
- Certification and verification of sustainability claims—including through Environmental, Social, and Governance (ESG) reporting frameworks, and life cycle analysis (LCA)—can help differentiate credible circular initiatives from greenwashing^57,95^.
- Resale platforms should avoid replicating fast-fashion norms, such as promoting urgency and novelty to stimulate frequent purchasing^37^.

Our findings also highlight the importance of psychological dynamics such as moral licensing and rebound effects. Marketing that overemphasizes consumer virtue may inadvertently enable overconsumption. More effective strategies would focus on encouraging moderation, repair, and extended garment use, rather than positioning resale as a self-contained solution.

### Limitations and future directions

These recommendations are based on U.S.-based behavioral data and do not assess the causal impact of specific policies or interventions. We do not evaluate the effects of existing frameworks such as the EU Strategy for Sustainable and Circular Textiles, nor do we analyze changes in fast-fashion market performance across jurisdictions. Future research should investigate the effects of policy tools—including taxation, durability labeling, and extended producer responsibility—across diverse regulatory and cultural settings. Additionally, further empirical work is needed to test whether the patterns of rebound and moral licensing observed here generalize to other countries and consumer populations.

### Limitations and interpretation of behavioral mechanisms

This study employs a cross-sectional survey design, which can identify behavioral patterns and associations but is limited in its ability to draw causal inferences regarding the psychological mechanisms that may underlie observed consumption behaviors, such as rebound effects or moral licensing^81^. While the survey captures correlations between and among sustainability-related attitudes, knowledge, and consumption patterns, it does not establish temporal or causal relationships. Accordingly, references to theoretical concepts such as the rebound effect and moral licensing are intended to guide interpretation and hypothesis development, rather than assert confirmed psychological or behavioral causation. This limitation is common in cross-sectional research designs, which are well-suited for identifying associations but not for establishing causal directionality^82^.

## Electronic Supplementary Material

Below is the link to the electronic supplementary material.

## Data Availability

The datasets generated and analyzed during the current study are not publicly available due to ethical restrictions and the need to protect participant confidentiality. Anonymized data will be made available by the corresponding author upon reasonable request, in accordance with the protocol approved by the Yale University Institutional Review Board (Protocol ID: 2000037433). The survey instrument will be available from the corresponding author upon reasonable request, rather than being included as an appendix.
