# Supplementary material for: Secondhand fashion consumers exhibit fast fashion behaviors despite sustainability narratives
Source: Sci Rep. 2025 Oct 7;15:34968. doi: 10.1038/s41598-025-19089-1 (PMC12504660; doi:10.1038/s41598-025-19089-1)
Supplement: Supplementary file 1 — Supplementary Material 1 [file 41598_2025_19089_MOESM1_ESM.docx]

Appendix B – Supplementary Tables

| Respondents by Age Group | % |
| --- | --- |
| 18-24 | 16.4% |
| 25-34 | 26.4% |
| 35-44 | 19.3% |
| 45-54 | 14.9% |
| 55-65 | 12.3% |
| 65+ | 10.7% |

**Supplementary Table 1**: Respondents by Age Group

| Respondents by Gender | % |
| --- | --- |
| Male | 44.3% |
| Female | 55.7% |

**Supplementary Table 2**: Respondents by Gender

| What is the highest level of education you have completed? | % |
| --- | --- |
| Less than a high school diploma | 3.2% |
| High school diploma or equivalent | 24.8% |
| Some college or associate degree | 28.2% |
| Bachelor’s degree | 29.5% |
| Master’s degree | 13.0% |
| Doctorate or professional degree | 1.3% |

**Supplementary Table 3**: Respondents by education

| What is your yearly household income? | % |
| --- | --- |
| Less than $25 000 | 21.8% |
| $25 000-$50 000 | 22.5% |
| $50 001-$100 000 | 33.0% |
| $100 001-$200 000 | 19.0% |
| $200 001-$250 000 | 1.7% |
| $250 001-$300 000 | 2.0% |

**Supplementary Table 4**: Respondents by yearly household income

| What is your employment situation? | % |
| --- | --- |
| Full-time employee | 40% |
| Part-time employee | 18% |
| Unemployed | 16% |
| Retirement | 14% |
| Student | 12% |

**Supplementary Table 5**: Respondents by employment situation

| What is your marital status? | % |
| --- | --- |
| Single | 45.7% |
| Single living with parents | 6.4% |
| Single with children | 4.5% |
| Living with partner | 26.0% |
| Living with partner with children | 17.4% |

**Supplementary Table 6**: Respondents by marital status

| To what extent are you politically involved? | % |
| --- | --- |
| Prefer not to disclose | 13.5% |
| Rarely engage in political activities or vote | 9.4% |
| Follow political news but do not actively participate | 37.6% |
| Regularly vote in elections but not actively involved in campaigns | 17.7% |
| Actively engaged in political activities and campaigns | 21.8% |

**Supplementary Table 7**: Respondents by politically involvement

| Average number of new fashion products purchased per month during the last year | |
| --- | --- |
| Less than 5 | 59.8% |
| Between 6 and 10 | 25.4% |
| Between 11 and 20 | 9.5% |
| Between 21 and 40 | 2.7% |
| over 40 items | 2.5% |

**Supplementary Table 8**: Average number of new fashion products purchased per month during the last year

| Average number of used fashion products purchased per month | |
| --- | --- |
| Less than 5 | 68.5% |
| Between 6 and 10 | 18.8% |
| Between 11 and 20 | 8.5% |
| Between 21 and 40 | 2.9% |
| over 40 items | 1.2% |

**Supplementary Table 9**: Average number of used fashion products purchased per month during the last year

| Looking back from 2020 to today, which of the following statements are true about your fashion purchasing habits? | Less | Same | More |
| --- | --- | --- | --- |
| Buying new clothes online | 19.7% | 42.3% | 38.0% |
| Buying used clothes | 28.5% | 47.9% | 23.7% |
| Buying new clothes in stores | 29.8% | 46.4% | 23.8% |
| Donating used clothes | 14.0% | 48.8% | 37.2% |
| Purchasing clothes from fashion chains that produce outside the USA (e.g., SHEIN, TEMU).4o | 32.8% | 42.5% | 24.6% |
| Attending clothes swap parties more frequently than before | 45.1% | 47.9% | 7.0% |
| Buying new clothes at cheaper prices | 10.5% | 48.7% | 40.8% |
| Selling used clothes | 35.0% | 47.1% | 17.9% |
| Buying clothes made in USA | 13.7% | 62.8% | 23.5% |
| buying clothes with sustainability as a significant consideration. | 15.2% | 58.7% | 26.1% |

**Supplementary Table 10**: Changes between 2020 and 2024

| Please mark the TWO most common reasons for you to get rid of clothes | |
| --- | --- |
| They are not in fashion anymore | 5.6% |
| They no longer fit my size | 40.8% |
| Desire to renew | 10.6% |
| I ordered online and they don’t look like I imagined | 8.7% |
| They are too worn out to be wear | 28.7% |
| They are no longer pleasing | 20.0% |
| They have gone out of fashion | 6.8% |
| I want to make room for new clothes | 19.0% |

**Supplementary Table 11**: Reasons to dispose of clothes

| For the most part, within how much time after purchasing them do you usually get rid of clothes? | |
| --- | --- |
| Up to a month from purchase | 14.2% |
| Between a month and six months | 10.3% |
| Between half a year and a year | 13.4% |
| Between one and five years | 32.5% |
| After more than five years | 29.6% |

**Supplementary Table 12**: Duration before disposing of clothes

| For the most part, when you get rid of clothes, their physical condition is: | |
| --- | --- |
| Brand new or like new | 7.9% |
| With light signs of use but still in good condition | 17.2% |
| Somewhat worn but still wearable | 28.8% |
| With noticeable stains and or tears | 27.8% |
| Suitable for reuse as rags | 18.3% |

**Supplementary Table 13**: The condition of clothes when they are disposed of

| Try to estimate, the percentage of clothes in your closet that you have purchased and not worn yet is: | |
| --- | --- |
| There are no items of clothing in my closet that I purchased and did not wear | 26.6% |
| There are less than ten items in my closet that I purchased but never wore | 33.1% |
| About a quarter of the clothes in my closet | 16.6% |
| a third of the clothes in my closet | 13.0% |
| More than half of the clothes in my closet | 10.6% |

**Supplementary Table 14**: Amount of unworn clothing in respondents’ closets

| When I am no longer interested in a garment I usually | 1 - Never | 2 | 3 | 4 | 5 - Several times each month |
| --- | --- | --- | --- | --- | --- |
| Pass it on to a friend or family who might wear it | 16.0% | 16.9% | 33.5% | 23.4% | 10.2% |
| Donate it to a second-hand store such as Goodwill, or Salvation Army | 9.6% | 12.4% | 32.6% | 30.0% | 15.3% |
| Place it in clothes recycling bins | 26.6% | 18.5% | 30.6% | 17.2% | 7.2% |
| Throw it in the trash | 50.1% | 20.2% | 18.0% | 8.0% | 3.6% |
| Sell it on a second-hand sales platform (stores/internet, fairs) | 41.7% | 19.0% | 20.5% | 13.2% | 5.5% |

**Supplementary Table 15**: common disposal methods

| Pearson’s r | Knowledge |
| --- | --- |
| UsedATTITUDE | .130** |
| Used_ACTIONS | -.095* |
| ConsiderSustain | -.107* |
| SustFashion_ATTIT | -.100* |
| * p<.05; ** p<.01 |  |

**Supplementary Table 16:** Demographic Profile of Consumer Clusters

|  |  | Cluster 1 | Cluster 2 |
| --- | --- | --- | --- |
| Gender | Male | 47% | 45% |
|  | Female | 53% | 55% |
|  |  | 100% | 100% |
| Household Income | Less than $ 25,000 | 25% | 17% |
|  | $ 25,000-$ 50,000 | 23% | 22% |
|  | $ 50,001-$ 100,000 | 30% | 37% |
|  | $ 100001 & above | 22% | 24% |
|  |  | 100% | 100% |
| Education | Less than college | 60% | 51% |
|  | At least college | 40% | 49% |
|  |  | 100% | 100% |
| Marital | Single | 62% | 49% |
|  | With partner | 38% | 51% |
|  |  | 100% | 100% |
| Political | Passive | 53% | 56% |
|  | Active | 47% | 44% |
|  |  | 100% | 100% |
| Age | 18-24 | 17% | 14% |
|  | 25-34 | 23% | 23% |
|  | 35-44 | 22% | 16% |
|  | 45-55 | 18% | 14% |
|  | 54-65 | 12% | 15% |
|  | 65+ | 8% | 18% |
|  |  | 100% | 100% |

**Supplementary Table 17**: Correlation between knowledge about fashion (as expressed in truth or falsehood) and the sustainability of fashion purchasing patterns

|  | Valid N | Missing cases | Mean | Median | Std. Deviation | Minimum | Maximum |
| --- | --- | --- | --- | --- | --- | --- | --- |
| NewMoney_TOTAL | 753 | 256 | 3.29 | 3.50 | 0.95 | 1 | 6 |
| UsedMoney_TOTAL | 515 | 494 | 4.45 | 4.75 | 1.12 | 1 | 6 |
| UsedATTITUDE | 737 | 272 | 3.25 | 3.25 | 0.78 | 1 | 5 |
| Used_ACTIONS | 726 | 283 | 2.81 | 2.87 | 0.79 | 1 | 5 |
| Returns | 490 | 519 | 2.96 | 3.14 | 0.71 | 1 | 5 |
| ConsiderPragmatic | 707 | 302 | 3.96 | 4.00 | 0.76 | 1 | 5 |
| ConsiderSustain | 707 | 302 | 3.11 | 3.00 | 0.91 | 1 | 5 |
| ConsiderConsume | 707 | 302 | 3.65 | 3.75 | 0.66 | 1 | 5 |
| Change.Sust | 731 | 278 | 2.75 | 3.00 | 0.92 | 1 | 5 |
| Change.New | 731 | 278 | 3.10 | 3.00 | 1.20 | 1 | 5 |
| Change.Local | 731 | 278 | 3.04 | 3.00 | 1.09 | 1 | 5 |
| Knowledge | 477 | 532 | 7.98 | 8.00 | 2.85 | 0 | 14 |
| SustFashion_ATTIT | 472 | 537 | 2.60 | 2.67 | 0.95 | 1 | 5 |
| ChangeIndex | 731 | 278 | 3.04 | 3.00 | 0.66 | 1 | 5 |
| FastNew | 767 | 242 | 3.73 | 3.81 | 0.66 | 1 | 5 |
| FastUsed | 515 | 494 | 4.18 | 4.33 | 0.78 | 1 | 5 |
| FootPrint | 726 | 283 | 2.80 | 2.79 | 0.42 | 1 | 5 |

**Supplementary Table 18:** Descriptive statistics and correlation matrix of all the indices

Appendix B – Legends

**Figure 1.** Behavioral clusters and their mean values across key variables.

The figure depicts the mean values of key variables for two behavioral clusters identified in the analysis. Cluster 1 (blue) represents 59% of respondents and is characterized by high consumption levels, frequent returns, shorter garment retention periods, and lesser sustainability knowledge and attitudes. Cluster 2 (orange) includes 41% of respondents, showing lower consumption levels, higher spending per item, and longer garment retention periods, coupled with greater sustainability knowledge. The variables on the x-axis include purchasing frequency, disposal behaviors, and environmental awareness metrics. The y-axis represents standardized mean values. These results highlight distinct patterns of consumption and sustainability engagement between the two clusters.

**Figure 2.** Principal Component Analysis (PCA) of demographic variables by cluster.

The PCA biplot illustrates the relationship between demographic variables and the two identified clusters. The first principal component (Dim1) explains 25.3% of the variance, and the second component (Dim2) explains 19.6%. Cluster 1 (blue) is strongly associated with younger individuals, particularly students and those employed part-time or full-time. Cluster 2 (orange) is more closely linked to older and retired individuals, as well as age group and gender variables. The arrows indicate the contributions of each variable to the PCA dimensions.

**Figure 3.** Indices of fashion consumption behaviors by age group.

The figure illustrates the mean scores of key fashion consumption indices across six age groups (18–24, 25–34, 35–44, 45–54, 55–65, and 65+). Younger respondents (18–24) demonstrated higher purchasing volumes and frequencies in both primary and secondary fashion markets, with 79% purchasing secondhand clothing as compared to 57% of respondents aged 65 and older. These patterns reflect frequent, lower-cost purchases and a higher tendency to own unworn items, indicating an extension of fast-fashion behaviors into the secondary market. Conversely, older respondents spent more per item, purchased fewer items, and retained clothing for longer periods, highlighting their focus on durability and longevity. The findings underscore generational differences in consumption behaviors, with younger individuals driving secondary market participation and potential rebound effects, while older individuals emphasize sustainable longevity.

**Figure 4.** Frequency, quantity, and total expenditure on new clothing by age group.

The figure shows the relationship between age and purchasing behaviors in the primary market, focusing on new clothing quantity, purchase frequency, and total expenditure (NewMoney_TOTAL). Younger respondents (18–24) exhibit higher purchase frequencies but lower spending per item, as reflected in the lower quantity and expenditure scores. In contrast, older respondents (65+) demonstrate lower purchasing frequency but significantly higher spending, indicating a preference for fewer, higher-quality items. A generalized linear model (Likelihood Ratio Chi-Square = 92.04, df = 11, p < 0.001) further revealed that individuals who purchase more new clothing score lower on sustainable secondhand purchasing behaviors, regardless of age. This finding underscores a trend in which high engagement in the primary market undermines sustainable practices across all age groups.

**Figure 5.** Indices of fashion consumption behaviors by income group.

The figure displays the mean scores for key fashion consumption indices across income groups, ranging from less than $25,000 to $250,001–$300,000. Higher-income respondents exhibited greater expenditure on new clothing (NewMoney_TOTAL) and higher levels of environmental knowledge. Despite this knowledge, their behaviors were less sustainable, as indicated by higher return rates, shorter garment retention periods, and a greater likelihood of discarding items in good condition. Lower-income groups demonstrated more sustainable consumption patterns but had lower environmental knowledge scores. These findings highlight a complementary relationship between primary and secondary markets for higher-income consumers, with increased consumption across both markets rather than substitutional behaviors.

**Figure 6.** Indices of fashion consumption behaviors by gender.

The figure illustrates the mean scores for key fashion consumption and sustainability indices for male and female respondents. Women demonstrated higher engagement in both primary and secondary markets, purchasing more frequently and in larger quantities than men. Women also showed more positive attitudes toward sustainability, greater environmental knowledge, and higher consideration for sustainability in purchasing decisions. Additionally, women were more likely to engage in sustainable disposal practices, such as repurposing clothing. However, their overall consumption patterns resulted in a larger environmental footprint, as indicated by higher footprint index scores. Men exhibited lower overall consumption levels and engagement with sustainability-related behaviors.

**Figure 7.** Indices of fashion consumption behaviors by level of political involvement.

The figure presents mean scores for key consumption and sustainability indices across four levels of political involvement: (1) rarely engaging in political activities or voting, (2) following political news without active participation, (3) regularly voting in elections without campaign involvement, and (4) actively engaging in political activities and campaigns. The relationship between political engagement and sustainable fashion behaviors is mixed. Politically engaged individuals spent less on new fashion overall, but differences in the FastNew index—which incorporates both quantity and frequency of new purchases—were less pronounced. Less politically active respondents scored higher on some sustainability-related indices, such as attitudes toward sustainable fashion, yet they also ranked highest on consumerist purchasing considerations and lowest on the FastUsed index. These respondents were more likely to own unworn new garments; to return items more frequently; and to dispose of clothing in good condition. These patterns suggest that lower political engagement may coincide with both constrained new fashion spending and behaviors that remain environmentally unsustainable.

**Table 1.** Distribution of respondents by age group.

The table summarizes the percentage of survey respondents across six age groups. The largest group is 25–34 years old, comprising 26.4% of respondents, followed by the 18–24 age group at 16.4%. The smallest group is 65 and older, representing 10.7% of the sample. This distribution reflects a diverse range of age groups, enabling analysis of generational differences in fashion consumption behaviors.

**Table 2.** Distribution of respondents by gender.

The table displays the percentage of survey respondents by gender. Female respondents constituted the majority of the sample at 55.7%, while male respondents accounted for 44.3%. This gender distribution allows for comparative analysis of fashion consumption behaviors and sustainability attitudes as between males and females.

**Table 3.** Distribution of respondents by education level.

The table presents the highest level of education completed by respondents. The largest group holds a bachelor’s degree (29.5%), followed by those with some college or an associate degree (28.2%) and those with a high school diploma or equivalent (24.8%). A smaller proportion of respondents reported having a master’s degree (13.0%), and very few completed a doctorate or professional degree (1.3%). Respondents with less than a high school diploma represented 3.2% of the sample. This distribution highlights a diverse range of educational backgrounds in the sample population.

**Table 4.** Distribution of respondents by yearly household income.

The table summarizes the yearly household income of respondents. The largest income group is $50,001–$100,000, comprising 33.0% of the sample, followed by $25,000–$50,000 at 22.5%, and less than $25,000 at 21.8%. Higher income groups are less represented, with 19.0% earning $100,001–$200,000, 2.0% earning $250,001–$300,000, and 1.7% earning $200,001–$250,000. This distribution reflects a diverse range of economic backgrounds within the sample.

**Table 5.** Distribution of respondents by employment situation.

The table displays the employment status of respondents. The largest group consists of full-time employees (40%), followed by part-time employees (18%) and unemployed individuals (16%). Respondents who are retired represent 14% of the sample, while students make up 12%. This distribution highlights the diversity in employment situations within the surveyed population.

**Table 6.** Distribution of respondents by marital status.

The table summarizes the marital status of respondents. The largest group is single (45.7%), followed by those living with a partner (26.0%) and those living with a partner with children (17.4%). Smaller proportions of respondents are single living with parents (6.4%) or single with children (4.5%). This distribution provides insight into the varied household and relationship dynamics within the sample population.

**Table 7.** Distribution of respondents by level of political involvement.

The table outlines the extent of political involvement among respondents. The largest group (37.6%) follows political news but does not actively participate, while 21.8% are actively engaged in political activities and campaigns. Respondents who regularly vote in elections without campaign involvement make up 17.7% of the sample. Smaller proportions rarely engage in political activities or vote (9.4%), and 13.5% prefer not to disclose their level of political involvement. This distribution highlights varying levels of political engagement within the sample population.

**Table 8.** Average number of new fashion products purchased per month during the last year.

The table displays the distribution of respondents based on the average number of new fashion products purchased per month in the past year. The majority of respondents (59.8%) purchased fewer than 5 items per month, while 25.4% purchased between 6 and 10 items. Smaller groups reported purchasing between 11 and 20 items (9.5%), 21 to 40 items (2.7%), and over 40 items (2.5%) per month.

**Table 9.** Average number of used fashion products purchased per month during the last year.

The table shows the distribution of respondents based on the average number of used fashion products purchased per month in the past year. Most respondents (68.5%) purchased fewer than 5 items per month, followed by 18.8% purchasing between 6 and 10 items. A smaller proportion purchased between 11 and 20 items (8.5%), 21 to 40 items (2.9%), and over 40 items (1.2%) per month.

**Table 10.** Changes in fashion purchasing habits between 2020 and 2024.

The table summarizes the changes in respondents’ fashion purchasing habits over the last four years. Key trends include an increase in online purchases of new clothes (38.0%) and donations of used clothes (37.2%). While the purchase of clothes at cheaper prices has also risen (40.8%), other sustainable practices, such as attending clothing swap parties (7.0%) and buying used clothes (23.7%), remain less common. The majority of respondents reported no change in buying habits for sustainable clothing considerations (58.7%) or clothes made in the USA (62.8%). These data reflect a complex shift in consumer behaviors, with both sustainable and unsustainable practices increasing.

**Table 11.** Reasons for disposing of clothes.

The table outlines the primary reasons respondents gave for getting rid of clothes. The most common reason was clothes no longer fitting their size (40.8%), followed by items being too worn out to wear (28.7%). Other common motivations included a desire to make room for new clothes (19.0%) and clothing no longer being pleasing (20.0%). Less frequently mentioned reasons include dissatisfaction with online purchases (8.7%), a desire for renewal (10.6%), and clothes being out of fashion (6.8%). These data highlight practical and emotional drivers of clothing disposal behaviors.

**Table 12.** Duration before disposing of clothes.

The table shows the time frame within which respondents typically dispose of clothing after purchase. The largest group (32.5%) reported keeping clothes for one to five years, followed by those retaining clothing for more than five years (29.6%). A smaller proportion disposed of items within a year (13.4%) or in less than six months (10.3%). Notably, 14.2% of respondents reported getting rid of clothes within a month of purchase, indicating a fast consumption-disposal cycle among some individuals.

**Table 13.** Physical condition of clothes at the time of disposal.

The table details the condition of clothing when respondents dispose of them. Most items are somewhat worn but still wearable (28.8%) or have noticeable stains or tears (27.8%). A significant portion of respondents dispose of clothes suitable for reuse as rags (18.3%) or items in good condition with light signs of use (17.2%). Only 7.9% of clothes were disposed of in brand new or like-new condition, suggesting diverse disposal practices influenced by clothing wear and tear.

**Table 14.** Amount of unworn clothing in respondents’ closets.

The table illustrates the estimated amount of clothing in respondents’ closets that they have never worn. The largest group (33.1%) reported having fewer than ten unworn items, while 26.6% claimed they had no unworn clothes. Smaller percentages indicated that about a quarter (16.6%), a third (13.0%), or more than half (10.6%) of their closets consisted of unworn items. These figures suggest variability in consumer habits regarding clothing use.

**Table 15.** Frequency of common clothing disposal methods.

The table shows how often respondents engage in various disposal methods, rated on a scale from 1 (never) to 5 (several times each month). Donating clothes to second-hand stores (e.g., Goodwill or Salvation Army) was the most frequent method, with 45.3% engaging in this practice at least monthly (rating 4 or 5). Passing clothes on to friends or family was also common, with 33.6% doing so monthly. Recycling bins and second-hand sales platforms were less frequently used. Roughly half of respondents—50.1%– reported never engaging in the least sustainable practice of throwing clothes in the trash. These data reflect diverse disposal behaviors and highlight opportunities to promote more sustainable practices.

**Table 16.** Demographic Profile of Consumer Clusters

Table 16 presents the demographic characteristics of the two consumer clusters identified in the study. While the clusters are relatively similar across gender and political engagement, some differences emerge in age, education, income, and marital status. Cluster 2 includes a higher proportion of older, more educated, and partnered individuals, as well as those with middle-to-upper income levels. These distinctions provide context for interpreting differences in consumption patterns and sustainability-related behaviors discussed in the main text.

**Table 17.** Correlation between fashion knowledge and sustainability in purchasing patterns.

The table presents Pearson’s r correlations between respondents’ fashion knowledge and sustainability-related attitudes and behaviors. A significant positive correlation (r=.130, p < .01) was found between knowledge and UsedATTITUDE, indicating that greater knowledge aligns with more positive attitudes toward second-hand clothing. However, small negative correlations were observed for Used_ACTIONS (r=-.095, p < .05), ConsiderSustain (r=-.107, p < .05), and SustFashion_ATTIT (r=-.100, p < .05), suggesting that while knowledge influences attitudes, it does not always translate to sustainable behaviors. Although statistically significant, these correlations are weak and can be viewed only as an indication for the complexity of the knowledge-behavior gap in sustainable fashion practices.

**Table 18.** Descriptive statistics for fashion consumption and sustainability indices.

The table provides descriptive statistics for all the indices used in the study, including the number of valid responses (Valid N), missing cases, mean, median, standard deviation, minimum, and maximum values. Key findings include the following:

- **NewMoney_TOTAL** (mean = 3.29) and **UsedMoney_TOTAL** (mean = 4.45) indicate differing spending patterns between new and used clothing markets.
- **Knowledge** had the highest variability, with a standard deviation of 2.85 and a range from 0 to 14.
- Sustainability-related indices, such as **ConsiderSustain** (mean = 3.11) and **Change.Sust** (mean = 2.75), suggest moderate levels of consideration for sustainable practices.
- **FastNew** (mean = 3.73) and **FastUsed** (mean = 4.18) highlight differences in consumption behaviors between new and used clothing markets. This table provides a comprehensive overview of respondents’ behaviors, attitudes, and knowledge regarding fashion consumption and sustainability practices.
